# Supplementary material for: Clinical and nutritional correlates of bacterial diarrhoea aetiology in young children: a secondary cross-sectional analysis of the ABCD trial
Source: BMJ Paediatr Open. 2024 Apr 11;8(1):e002448. doi: 10.1136/bmjpo-2023-002448 (PMC11015214; doi:10.1136/bmjpo-2023-002448)
Supplement: Supplementary data [file bmjpo-2023-002448supp001.pdf]

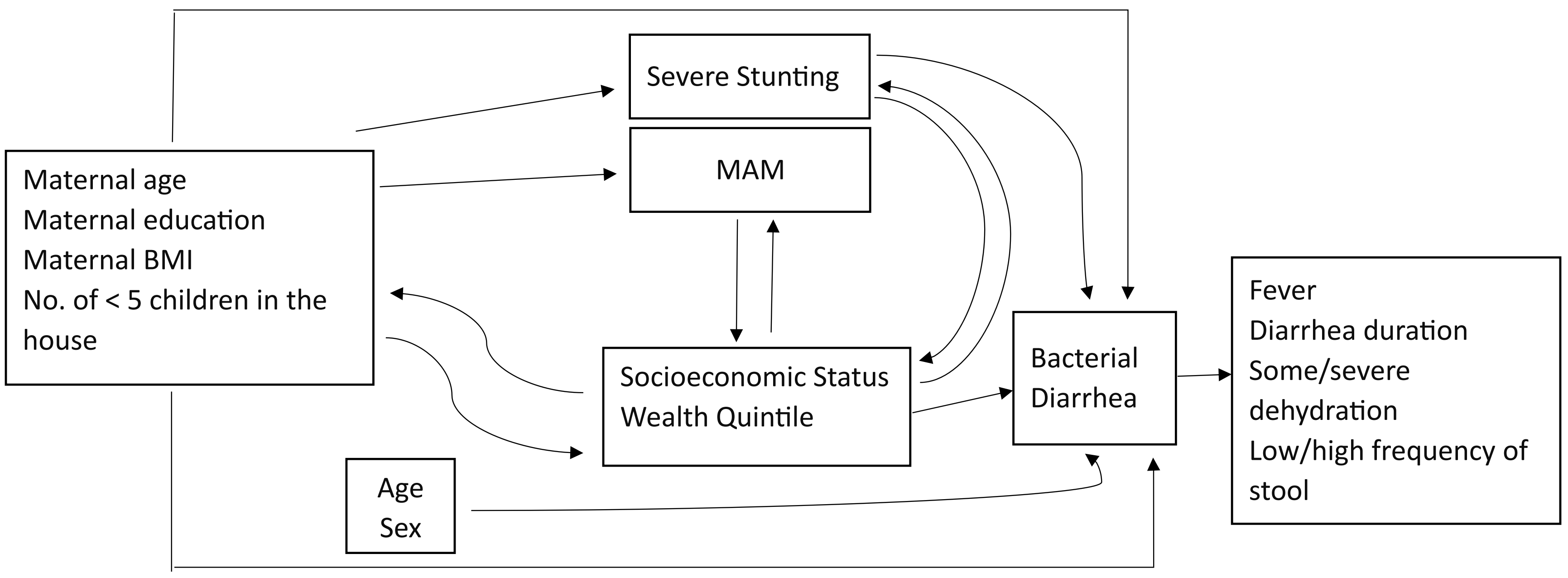

Supplementary Figure 1: A directed acyclic graph (DAG) depicting the relationship between the exposure, outcome and confounding variables that are included in this analysis.
