# Supplementary material for: Clinical and nutritional correlates of bacterial diarrhoea aetiology in young children: a secondary cross-sectional analysis of the ABCD trial
Source: BMJ Paediatr Open. 2024 Apr 11;8(1):e002448. doi: 10.1136/bmjpo-2023-002448 (PMC11015214; doi:10.1136/bmjpo-2023-002448)
Supplement: Supplementary data [file bmjpo-2023-002448supp002.pdf]

Supplementary table 1: Clinical and nutritional unadjusted odds ratios of likely bacterial etiology of diarrhea among 2-23-month-old children with moderate-to-severe diarrhea

	Bacterial etiology			Escherichia coli encoding heat-stable toxin etiology			Shigella etiology		
**Variable**	**Proportion n/N (%)**	**Unadjusted odds ratio (95% CI)**	**p-value**	**Proportion n/N (%)**	**Unadjusted odds ratio (95% CI)**	**p-value**	**Proportion n/N (%)**	**Unadjusted odds ratio (95% CI)**	**p-value**
**Moderate to severe diarrhea defining characteristics**									
**Severe stunting only**	94/417 (22.5%)	Ref	-	40/417 (9.6%)	Ref	-	52/417 (12.5%)	Ref	-
**Some / severe dehydration only**	764/2,832 (27.0%)	1.27 (1.00,1.63)	0.06	378/2815 (13.4%)	1.46 (1.05,2.09)	0.030	305/2832 (10.8%)	0.85 (0.62, 1.17)	0.30
**MAM only**	675/2,228 (30.3%)	1.47 (1.16,1.90)	0.93	319/ 2,227 (14.3%)	1.58 (1.13,2.26)	0.01	320/2224 (14.4%)	1.18 (0.87,1.63)	0.31
**MAM and some /severe dehydration**	209/626 (33.4%)	1.70 (1.28,2.27)	0.002	81/623 (13.0%)	1.41 (0.95,2.12)	0.09	90/626 (14.4%)	1.18 (0.82,1.71)	0.38
**MAM and severe stunting**	109/408 (26.7%)	1.24 (0.90,1.70)	0.19	45/408 (11.0%)	1.17 (0.75,1.84)	0.50	63/408 (15.4%)	1.28 (0.86,1.91)	0.22
**Some/severe dehydration and severe stunting**	22/95 (23.2%)	1.02 (0.59, 1.71)	0.93	14/95 (14.7%)	1.63 (0.82,3.07)	0.15	5/95 (5.3%)	0.39 (0.13,0.92)	0.051
**MAM, some/severe dehydration and severe stunting**	20/84 (23.8%)	1.06 (0.60,1.81)	0.84	12/84 (14.3%)	1.57 (0.76,3.06)	0.20	10/84 (11.9%)	0.95 (0.44, 1.88)	0.89
**Fever**									
**No**	1,646/5,884 (28.0%)	Ref	-	767/5,865 (13.1%)	Ref	-	736/5,883 (12.5%)	Ref	-
**Yes**	248/808 (30.7%)	1.14 (0.97,1.34)	0.11	122/806 (15.1%)	1.19 (0.96. 1.45)	0.11	109/808 (13.5%)	1.09 (0.88, 1.35)	0.43
**Duration of diarrhea (excluding day of enrollment)**									
**Diarrhea (0-6 days)**	1,781/6,328 (28.1%)	Ref	-	846/6,308 (13.4%)	Ref	-	783/6,327 (12.4%)	Ref	-
**Prolonged duration (7-14 days)**	113/364 (31.0%)	1.15 (0.91,1.44)	0.23	43/363 (11.8%)	0.87 (0.62, 1.19)	0.39	62/364 (17.0%)	1.45 (1.09,1.92)	0.010
**Frequency of diarrhea in the past 24 hours**									
**Low frequency (3-6 stools)**	912/3,536 (25.8%)	Ref	-	433/3,521 (12.3%)	Ref	-	406/3,535 (11.5%)	Ref	-
**High frequency (>6 loose stools)**	982/3,156 (31.1%)	1.30 (1.17, 1.45)	<0.001	456/3,150 (14.5%)	1.21 (1.05, 1.39)	0.009	439/3,156 (13.9%)	1.25 (1.08,1.44)	0.003
