# Supplementary material for: Clinical and nutritional correlates of bacterial diarrhoea aetiology in young children: a secondary cross-sectional analysis of the ABCD trial
Source: BMJ Paediatr Open. 2024 Apr 11;8(1):e002448. doi: 10.1136/bmjpo-2023-002448 (PMC11015214; doi:10.1136/bmjpo-2023-002448)
Supplement: Supplementary data [file bmjpo-2023-002448supp003.pdf]

Supplementary table 2: Clinical and nutritional correlates with likely bacterial diarrheal etiology with no coinfection including enterotoxigenic Escherichia coli (E.coli) encoding heat-stable toxin (ST-ETEC) and Shigella diarrheal etiology with no coinfection in 2-23-month-old children with moderate-to-severe diarrhea adjusted for factors.

	**Bacterial etiology (no coinfection)**			***Escherichia coli* encoding heat-stable toxin etiology (no coinfection)**			***Shigella* etiology (no coinfection)**		
	**Proportion n/N (%)**	**Adjusted[1] odds ratio (95% CI)**	**p-value**	**Proportion n/N (%)**	**Adjusted[1] odds ratio (95% CI)**	**p-value**	**Proportion n/N (%)**	**Adjusted[1] odds ratio (95% CI)**	**p-value**
**Moderate to severe diarrhea defining characteristics**									
**Severe stunting only**	65/417 (15.6%)	Ref.	-	18/417 (4.3%)	Ref.	-	35/417 (8.4%)	Ref.	-
**Some /severe dehydration only**	464/2,336 (16.6%)	1.34 (0.96, 1.88)	0.088	156/2,824 (5.5%)	1.36 (0.79, 2.52)	0.30	150/2,829 (5.3%)	0.66 (0.42, 1.05)	0.074
**MAM only**	382/2,2224 (17.2%)	1.18 (0.86,1.65)	0.31	116/2,221 (5.2%)	1.28 (0.74, 2.37)	0.40	154/2,223 (6.9%)	0.95 (0.61, 1.50)	0.81
**MAM and some /severe dehydration**	107/623 (17.2%)	1.38 (0.94, 2.02)	0.10	28/625 (4.5%)	1.23 (0.63, 2.47)	0.55	30/626 (4.8%)	0.64 (0.36, 1.14)	0.13
**MAM and severe stunting**	71/407 (17.4%)	0.91 (0.59, 1.39)	0.66	20/407 (4.9%)	1.05 (0.50, 2.22)	0.89	33/407 (8.1%)	0.61 (0.33, 1.11)	0.11
**Some/severe dehydration and severe stunting**	8/95 (8.4%)	0.70 (0.29, 1.47)	0.37	5/95 (5.3%)	1.38 (0.43, 3.75)	0.55	2/95 (2.1%)	0.28 (0.04, 0.98)	0.09
**MAM, some/severe dehydration, and severe stunting**	14/84 (16.7%)	0.75 (0.31, 1.60)	0.49	6/84 (7.1%)	1.40 (0.39, 4.05)	0.57	3/84 (3.6%)	0.29 (0.05, 1.01)	0.098
**Fever**									
**No**	986/5,845 (16.9%)	Ref.	-	312/5,870 (5.3%)	Ref	-	364/5,880 (6.2%)	Ref	-
**Yes**	124/803 (15.4%)	0.88 (0.70,1.10)	0.29	36/806 (4.5%)	0.79 (0.53, 1.15)	0.24	43/763 (5.3%)	0.88 (0.60,1.27)	0.52
**Duration of diarrhea (excluding day of enrollment)**									
**Diarrhea (0-6 days)**	1,036/ 6,285 (16.5%)	Ref.	-	329/6,314 (5.2%)	Ref	-	32/363 (8.8%)	Ref	-
**Prolonged duration (7-14 days)**	74/363 (20.4%)	1.22 (0.90,1.64)	0.19	19/362 (5.2%)	1.05 (0.60,1.73)	0.85	375/6,323 (5.9%)	1.19 (0.75,1.83)	0.44
**Frequency of diarrhea in the past 24 hours**									
**Low frequency (3-6 stools)**	534/3,508 (15.2%	Ref.	-	195/3,528 (5.5%)	Ref	-	202/3,534 (5.7%)	Ref	-
**High frequency (>6 loose stools)**	576/3,140 (18.3%)	1.23 (1.05,1.45)	0.0091	153/3,150 (4.9%)	0.99 (0.76, 1.28)	0.94	205/3,152 (6.5%)	1.32 (1.04,1.68)	0.025

[1] Adjusted for: child age, child sex, household wealth quintile, maternal age, maternal education, country of enrolment, rotavirus vaccination status, maternal BMI, total number of children under age 5 year in the household, defining characteristic of moderate to severe diarrhea, fever, duration of diarrhea, and high vs. low frequency of stools in past 24h.
