# Supplementary material for: Clinical and nutritional correlates of bacterial diarrhoea aetiology in young children: a secondary cross-sectional analysis of the ABCD trial
Source: BMJ Paediatr Open. 2024 Apr 11;8(1):e002448. doi: 10.1136/bmjpo-2023-002448 (PMC11015214; doi:10.1136/bmjpo-2023-002448)
Supplement: Supplementary data [file bmjpo-2023-002448supp004.pdf]

Supplementary table 3: Clinical and nutritional correlates with bacterial diarrheal etiology with no coinfection including enterotoxigenic Escherichia coli (E.coli) encoding heat-stable toxin (ST-ETEC) and Shigella diarrheal etiology with no coinfection in 2-23-month-old children with moderate-to-severe diarrhea.

	**Bacterial etiology (no coinfection)**			**Escherichia coli encoding heat-stable toxin etiology (no coinfection)**			**Shigella etiology (no coinfection)**		
**Variable**	**Proportion n/N (%)**	**Unadjusted odds ratio (95% CI)**	**p-value**	**Proportion n/N (%)**	**Unadjusted odds ratio (95% CI)**	**p-value**	**Proportion n/N (%)**	**Unadjusted odds ratio (95% CI)**	**p-value**
**Moderate to severe diarrhea defining characteristics**									
**Severe stunting only**	65/417 (15.6%)	Ref	-	18/417 (4.3%)	Ref.	-	35/417 (8.4%)	Ref	-
**Some / severe dehydration only**	464/2,336 (16.6%)	1.08 (0.82,1.44)	0.61	156/2,824 (5.5%)	1.30 (0.81,2.21)	0.31	150/2,829 (5.3%)	0.61 (0.42,0.91)	0.01
**MAM only**	382/2,2224 (17.2%)	1.12 (0.85, 1.51)	0.43	116/2,221 (5.2%)	1.22 (0.75,2.09)	0.45	154/2,223 (6.9%)	0.81 (0.56, 1.21)	0.28
**MAM and some /severe dehydration**	107/623 (17.2%)	1.12 (0.80,1.58)	0.50	28/625 (4.5%)	1.04 (0.57,1.94)	0.90	30/626 (4.8%)	0.55 (0.33,0.91)	0.020
**MAM and severe stunting**	71/407 (17.4%)	1.14 (0.79, 1.65)	0.48	20/407 (4.9%)	1.14 (0.59, 2.21)	0.69	33/407 (8.1%)	0.96 (0.58,1.58)	0.87
**Some/severe dehydration and severe stunting**	8/95 (8.4%)	0.50 (0.21, 1.02)	0.076	5/95 (5.3%)	1.23 (0.39, 3.18)	0.69	2/95 (2.1%)	0.24 (0.04, 0.79)	0.049
**MAM, some/severe dehydration and severe stunting**	14/84 (16.7%)	1.08 (0.56, 1.99)	0.80	6/84 (7.1%)	1.71 (0.60, 4.21)	0.27	3/84 (3.6%)	0.40 (0.09, 1.16)	0.14
**Fever**									
**No**	986/5,845 (16.9%)	Ref	-	312/5,870 (5.3%)	Ref	-	364/5,880 (6.2%)	Ref	-
**Yes**	124/803 (15.4%)	0.90 (0.73, 1.10)	0.31	36/806 (4.5%)	0.83 (0.58, 1.17)	0.31	43/763 (5.3%)	0.85 (0.61,1.17)	0.34
**Duration of diarrhea (excluding day of enrollment)**									
**Diarrhea (0-6 days)**	1,036/ 6,285 (16.5%)	Ref	-	329/6,314 (5.2%)	Ref	-	32/363 (8.8%)	Ref	-
**Prolonged duration (7-14 days)**	74/363 (20.4%)	1.30 (0.99,1.68)	0.05	19/362 (5.2%)	1.01 (0.61, 1.58)	0.98	375/6,323 (5.9%)	1.53 (1.03,2.20)	0.03
**Frequency of diarrhea in the past 24 hours**									
**Low frequency (3-6 stools)**	534/3,508 (15.2%	Ref	-	195/3,528 (5.5%)	Ref	-	202/3,534 (5.7%)	Ref	-
**High frequency (>6 loose stools)**	576/3,140 (18.3%)	1.25 (1.10,1.42)	<0.001	153/3,150 (4.9%)	0.87 (0.70, 1.08)	0.22	205/3,152 (6.5%)	1.15 (0.94, 1.40)	0.18
