# Supplementary material for: Clinical and nutritional correlates of bacterial diarrhoea aetiology in young children: a secondary cross-sectional analysis of the ABCD trial
Source: BMJ Paediatr Open. 2024 Apr 11;8(1):e002448. doi: 10.1136/bmjpo-2023-002448 (PMC11015214; doi:10.1136/bmjpo-2023-002448)
Supplement: Author's manuscript [file bmjpo-2023-002448.draft_revisions.pdf]

BMJ Paediatrics Open

BMJ Paediatrics Open is committed to open peer review. As part of this commitment we make the peer review history of every article we publish publicly available.

When an article is published we post the peer reviewers' comments and the authors' responses online. We also post the versions of the paper that were used during peer review. These are the versions that the peer review comments apply to.

The versions of the paper that follow are the versions that were submitted during the peer review process. They are not the versions of record or the final published versions. They should not be cited or distributed as the published version of this manuscript.

BMJ Paediatrics Open is an open access journal and the full, final, typeset and author-corrected version of record of the manuscript is available on our site with no access controls, subscription charges or pay-per-view fees (http://bmjpaedsopen.bmj.com).

If you have any questions on BMJ Paediatrics Open's open peer review process please email info.bmjpo@bmj.com

BMJ Paediatrics Open

Clinical and nutritional correlates of bacterial diarrhea etiology in young children: A secondary cross-sectional analysis of the ABCD trial

Journal:	*BMJ Paediatrics Open*
Manuscript ID	bmjpo-2023-002448
Article Type:	Original research
Date Submitted by the Author:	13-Dec-2023

Complete List of Authors:	Somji, Sarah; Tampere University, Center for Child, Adolescent, and Maternal Health Research, Faculty of Medicine and Health Technology; Muhimbili University of Health and Allied Sciences, Department of Epidemiology<br>Ashorn, Per; Tampere University, Center for Child, Adolescent, and Maternal Health Research, Faculty of Medicine and Health Technology<br>Manji, Karim; Muhimbili University of Health and Allied Sciences, Pediatrics<br>Ahmed, Tahmeed; International Centre for Diarrhoeal Disease Research Bangladesh, Nutrition and Clinical Sciences Division<br>Chisti, Md.; International Centre for Diarrhoeal Disease Research Bangladesh, Nutrition and Clinical Services Devision<br>Dhingra, Usha; Center for Public Health Kinetics<br>Sazawal, Sunil; Centre for Public Health Kinetics (CPHK)<br>Singa, Benson; Kenya Medical Research Institute, Center for Clinical Research<br>Walson, Judd L.; University of Washington, Department of Global Health; University of Washington, Department of Epidemiology<br>Pavlinac, Patricia; University of Washington, Department of Global Health; University of Washington, Department of Epidemiology<br>Bar-Zeev, Naor; Johns Hopkins Bloomberg School of Public Health, International Vaccine Access Center<br>Houpt, Eric; University of Virginia, Department of Medicine, Infectious Diseases<br>Dube, Queen; Queen Elizabeth Central Hospital, Department of Pediatrics<br>Kotloff, Karen; University of Maryland Baltimore, Center for Vaccine Development and Global Health; University of Maryland, Department of Pediatrics, Center for Vaccine Development and Global Health<br>Sow, Samba; Centre pour le Developpement des Vaccins Mali<br>Yousafzai, Mohammad Tahir; The Aga Khan University<br>Qamar, Farah; The Aga Khan University, Department of Pediatrics and Child Heath<br>Bahl, Rajiv ; World Health Organization, Department of Maternal, Child, and Adolescent Health and Aging<br>De Costa, Ayesha; World Health Organization, Department of Maternal, Child, and Adolescent Health and Aging<br>Simon, Jonathon; World Health Organization, Department of Maternal,

Child, and Adolescent Health and Aging
Sudfeld, Christopher; Harvard University T H Chan School of Public Health, Department of Global Health and Population and Nutrition
Duggan, Christopher; Boston Children's Hospital, Division of Gastroenterology, Hepatology and Nutrition; Harvard University T H Chan School of Public Health, Department of Global Health and Population and Nutrition
Study Group, ABCD; ABCD Study Group

Keywords: Epidemiology, Infant

SCHOLARONE™
Manuscripts

I, the Submitting Author has the right to grant and does grant on behalf of all authors of the Work (as defined in the below author licence), an exclusive licence and/or a non-exclusive licence for contributions from authors who are: i) UK Crown employees; ii) where BMJ has agreed a CC-BY licence shall apply, and/or iii) in accordance with the terms applicable for US Federal Government officers or employees acting as part of their official duties; on a worldwide, perpetual, irrevocable, royalty-free basis to BMJ Publishing Group Ltd ("BMJ") its licensees and where the relevant Journal is co-owned by BMJ to the co-owners of the Journal, to publish the Work in this journal and any other BMJ products and to exploit all rights, as set out in our licence.

The Submitting Author accepts and understands that any supply made under these terms is made by BMJ to the Submitting Author unless you are acting as an employee on behalf of your employer or a postgraduate student of an affiliated institution which is paying any applicable article publishing charge ("APC") for Open Access articles. Where the Submitting Author wishes to make the Work available on an Open Access basis (and intends to pay the relevant APC), the terms of reuse of such Open Access shall be governed by a Creative Commons licence – details of these licences and which Creative Commons licence will apply to this Work are set out in our licence referred to above.

Other than as permitted in any relevant BMJ Author's Self Archiving Policies, I confirm this Work has not been accepted for publication elsewhere, is not being considered for publication elsewhere and does not duplicate material already published. I confirm all authors consent to publication of this Work and authorise the granting of this licence.

Confidential: For Review Only

Clinical and nutritional correlates of bacterial diarrhea etiology in young children: A secondary cross-sectional analysis of the ABCD trial

Sarah Somji[1,2], Per Ashorn[1], Karim Manji [3], Tahmeed Ahmed[4], Mohammod Jobayer Chisti[4], Usha Dhingra[5], Sunil Sazawal[5], Benson Singa[6], Judd L Walson[7,8,9], Patricia Pavlinac[7,8], Naor Bar-Zeev[10], Eric Houpt[11], Queen Dube[12], Karen Kotloff[13,14], Samba Sow[15], Mohammad Tahir Yousafzai[16], Farah Naz Qamar[16], Rajiv Bahl[17], Ayesha De Costa[17], Jonathon Simon[17], Christopher Sudfeld*[18], Christopher P. Duggan*[18,19] and the ABCD Study Group**

*contributed equally

** Listed in the acknowledgements

Corresponding Author: Sarah Somji, PhD Student, Center for Child, Adolescent, and Maternal Health Research, Faculty of Medicine and Health Technology, Tampere University and Tampere University Hospital, Tampere, Finland; sarahsomji@gmail.com and Department of Epidemiology, Muhimbili University of Health and Allied Sciences, Dar-es-Salaam, Tanzania

Author affiliations:

1. Center for Child, Adolescent, and Maternal Health Research, Faculty of Medicine and Health Technology, Tampere University and Tampere University Hospital, Tampere, Finland
2. Department of Epidemiology, Muhimbili University of Health and Allied Sciences, Dar-es-Salaam, Tanzania

Confidential: For Review Only

3. Department of Pediatrics, Muhimbili University of Health and Allies Sciences, Dar-es-Salaam, Tanzania
4. Nutrition and Clinical Services Division, International Centre for Diarrheal Disease Research, Bangladesh, Dhaka, Bangladesh
5. Center for Public Health Kinetics (CPHK), New Delhi, India
6. Kenya Medical Research Institute (KEMRI), Center for Clinical Research, Nairobi, Kenya
7. Department of Global Health, University of Washington, Seattle USA
8. Department of Epidemiology, University of Washington, Seattle USA
9. Department of Pediatrics and Medicine (Infectious Diseases), University of Washington, Seattle, USA
10. International Vaccine Access Center, Johns Hopkins Bloomberg School of Public Health, Baltimore, Maryland
11. Department of Medicine, Infectious Diseases ; International Health, University of Virginia, Charlottesville, USA
12. Department of Pediatrics, Queen Elizabeth Central Hospital, Blantyre, Malawi
13. Center for Vaccine Development and Global Health, University of Maryland School of Medicine, Baltimore
14. Department of Pediatrics, Center for Vaccine Development and Global Health, University of Maryland School of Medicine, Baltimore, MD, USA
15. Centre pour le Développement des Vaccines, Bamako, Mali
16. Department of Pediatrics and Child Heath, Aga Khan University, Karachi, Pakistan

Confidential: For Review Only

17. Department of Maternal, Child, and Adolescent Health and Aging, World Health Organization, Geneva, Switzerland
18. Departments of Global Health and Population and Nutrition, Harvard T.H. Chan School of Public Health, Boston, Massachusetts
19. Division of Gastroenterology, Hepatology and Nutrition, Boston Children's Hospital, Department of Nutrition, Harvard T.H. Chan School of Public Health, Boston, Massachusetts

Keywords: bacterial diarrhea, molecular diagnostics, pediatric diarrhea, nutritional status

Confidential: For Review Only

ABSTRACT

Objective: The objective was to assess the association between nutritional and clinical characteristics and qPCR-diagnosis of bacterial diarrhea in a multicenter cohort of children under two years of age with moderate to severe diarrhea (MSD).

Design: A secondary cross-sectional analysis of baseline data collected from the AntiBiotics for Children with Diarrhea trial (NCT03130114).

Patients: Children with MSD (defined as ≥ 3 loose stools within 24 hours and presenting with at least one of the following: some/severe dehydration, moderate acute malnutrition (MAM), or severe stunting) enrolled in the ABCD trial and collected stool sample.

Study period: June 2017 to July 2019

Interventions: None

Main outcome measures: Likely bacterial etiology of diarrhea. Secondary outcomes included specific diarrhea etiology.

Results: A total of 6,692 children with MSD had qPCR results available and 28% had likely bacterial diarrhea etiology. Compared to children with severe stunting, children with MAM (adjusted odds ratio (aOR) [95% confidence intervals] 1.56 [1.18,2.08]), some/severe dehydration (aOR [95% CI] 1.66 [1.25,2.22]) or both (aOR [95% CI] 2.21 [1.61,3.06]), had higher odds of having likely bacterial diarrhea etiology. Similar trends were noted for ST-ETEC etiology. Clinical correlates including fever and prolonged duration of diarrhea were not associated with likely bacterial etiology; children with more than 6 stools in the previous 24h had higher odds of likely bacterial diarrhea (aOR [95% CI] 1.20 [1.05,1.36]) compared to those with fewer stools.

Confidential: For Review Only

Conclusion: The presence of MAM, dehydration or high stool frequency may be helpful in identifying children with MSD who might benefit from antibiotics.

What is already known on this topic

- Nutritional status has a bidirectional relationship with diarrhea incidence and severity.
- Clinical factors such as dehydration and prolonged duration are known to be a risk factor for severe diarrhea.
- Limited data exist on the relationship between nutritional and clinical status and specific infectious etiology of childhood diarrhea.

What this study adds

- Moderate acute malnutrition, dehydration, or both were found to have increased odds of likely bacterial etiology as compared to severe stunting among children with moderate to severe diarrhea.
- Children with more than 6 loose or watery stools in the 24h before presentation are at higher odds of likely bacterial diarrhea compared to those with lower stool frequency.

How this study might affect research, practice or policy

- Moderately malnourished or dehydrated children with diarrhea (or those with both) have higher you

Confidential: For Review Only

INTRODUCTION

Diarrhea causes over 400,000 deaths annually and strategies to reduce this burden are informed by studies of diarrhea etiology (1).Bacterial pathogens including enterotoxigenic *Escherichia coli (E.coli)* encoding heat stable toxin (ST-ETEC), *Shigella* are commonly associated with childhood diarrhea (2,3). The World Health Organization (WHO) recommends antibiotics for diarrhea only if there is blood in stool (dysentery) or in suspected cases of cholera(4). Increasing evidence, however, suggest that acute watery diarrhea can have bacterial (*Shigella* or enteroinvasive *Escherichia coli* EIEC) etiology as seen in an age-stratified case-control global enteric multicenter study (GEMs). This study enrolled cases who had moderate to severe diarrhea in children under 5 and noted in their collected stool samples that 40.3% *Shigella* spp or EIEC diarrhea attributed cases were non-dysenteric(3). Whether children with diarrhea of bacterial etiology can be identified, may have implications for antibiotic treatment recommendations.

While the relationship between nutritional status and incidence of diarrheal disease is well established, few studies have examined the relationship between child anthropometric and clinical characteristics with the specific etiology of diarrhea.Among 2 to 5-year-old Bangladeshi children with diarrhea, those with malnutrition (defined as weight-for-age Z-score < -2) were more likely to present with specific parasitic and bacterial enteropathogens specifically enterotoxigenic *Escherichia coli* (ETEC) (5). Another study in Kenya noted that malnourished children were more likely to have moderate to severe diarrhea (MSD) due to enteroaggregative *Escherichia coli* (EAEC) but in another study in Bangladesh in a similar cohort, similar children were likely to present with *Shigella*(6,7). While these studies suggest that acute malnutrition may be an important risk factor for bacterial causes of diarrhea, they are notable for being performed at single sites, among children with either low-risk diarrhea or in hospitalized children, or collected with traditional bacterial culture methods, and/or for a limited number of pathogens. We recently completed a large, multicenter clinical trial of antibiotics for moderate-to-severe diarrhea in

Confidential: For Review Only

children ages 2-23 months (8,9). This cohort provided an opportunity to better understand whether nutritional status and other clinical characteristics were associated with specific pathogens causing diarrhea across several countries. Participants in the trial presented with moderate-to-severe diarrhea and were assessed using molecular detection tools for several types of enteropathogens. We hypothesized that among children with MSD, those with MAM would be more likely to have a bacterial etiology.

METHODS

Participants

We studied a subsample of participants from the ABCD trial, a randomized, blinded, placebo-controlled, multicountry trial designed to evaluate whether azithromycin would reduce all-cause mortality in young children with undernutrition and MSD (8,9). The trial was implemented in Bangladesh, India, Kenya, Malawi, Mali, Pakistan and Tanzania between June 2017 and July 2019 in MSD children 2-23 months of age. For the purpose of this study, MSD was defined as a history of acute watery diarrhea (≥ 3 loose/watery stool in the past 24 hours) for <14 days with one of more of the following: the presence of some/severe dehydration; MAM (defined as weight-for-length z-score [WLZ] ≤ -2 and > -3 (or mid-upper arm circumference [MUAC] ≥ 115 mm and < 125 mm for children over 6 months)); or severe stunting (length-for-age z-score [LAZ] < -3). Exclusion criteria included receipt of antibiotics currently/ in previous 14 days; any allergy or contraindication to azithromycin; clinical suspicion of *V. cholerae* infection; previous/current enrollment of the child in this/any interventional trial; enrollment of a sibling or another child in the same household; or living at a distance from the enrolment center that would prevent direct observation on days 2 and 3. After written informed consent was obtained, participants were randomized to receive either a three day course of azithromycin or placebo (9). The subsample were participants identified randomly, at baseline with equal numbers from all sites.

Confidential: For Review Only

Exposure and Confounding Variables

MSD defining characteristics were the primary exposure of interest and included severe stunting, MAM, some/severe dehydration, or a combination of either of the two or all three characteristics. All anthropometric measurements were performed by 2 independant trained assessors according to WHO guidelines (8). Weight was measured using an electronic scale to the closest $\pm$ 10 g, length was obtained using a length board to the nearest 0.1 cm and MUAC was obtained using a non-stretchable tape to the nearest 0.1 cm. Study physicians measured and recorded the temperature using a digital thermometer at screening. Fever was calculated as temperature $\geq 37.5^{o}$C. Diarrhea duration and diarrhea frequency in the past 24 hours was recorded from mothers recall. Numerous confounding variables were collected at baseline using standardized questionnaires across all sites. A wealth quintile variable was created using country specific wealth distribution as reported in each country's most recent Demographic and Health Survey (10).

Outcome Variables

The primary outcome variable was likely bacterial diarrhea etiology assessed by qPCR on a baseline stool sample. Secondary outcomes included specific bacterial diarrhea etiology (likely ST-ETEC or *Shigella)*. Stool or rectal swab samples were tested by qPCR with a customized 85-target TaqMan array card to determine a cycle threshold (CTt) value and is detailed elsewhere (3). Pathogen-specific cutoffs were developed to assign likely diarrhea etiology based on the quantity of pathogen DNA/RNA in the stool sample (i.e., pathogen burden). These cut-offs(11) were obtained using adapted statistical models from two previously performed large multisite diarrhea studies: the seven-site Global Enteric Multicenter Study (GEMS) and the eight-site Malnutrition and the Consequences for Child Health and Development (MAL-ED) cohort study (3,12). Each cut-off was calculated by taking the median quantity-specific odds ratio from site specific models from previous studies. Then, the episode-specific attributable fraction (AFe) was

Confidential: For Review Only

calculated. Finally, a LOESS regression was fit and the highest Ct value with $\geq$ 0.5 Afe value was picked as the cut-off for each pathogen (13,14).

These Ct value cut-offs were considered “likely” (diarrhea associated) etiology. For this analysis, Ct values greater than these cut-offs were considered as not diarrhea associated etiology. For the current analysis, specimens assessed as “likely” were compared with the other category. Ascribing etiology to “likely” with these cut-offs has been previously performed (15). Bacteria assessed for etiology included *Campylobacter*, tEPEC, ST-ETEC, *Salmonella, Shigella, and V.cholerae*. *Shigella* and ST-ETEC were chosen as the main specific etiologies since they comprised of about 84% of all bacterial enteropathogens. Due to logistical and budgetary constraints, stool/rectal swabs from approximately the first 1,000 participants at each site were collected. Whole stool sample or flocked rectal swab were stored at -80˚C until analysis.

Statistical Analysis

We conducted a cross-sectional analysis of baseline data from the ABCD trial. Logistic regressions were used to examine the association of clinical and anthropometric characteristics, including MSD defining characteristics: fever, diarrhea duration, and stool frequency, with likely bacterial diarrhea etiology. For the variable MSD defining characteristic, the presence of severe stunting alone was used as the reference category since participants with severe stunting had the lowest prevalence of likely bacterial diarrhea. Some previous studies also suggest that stunting is not associated with presence of specific pathogens in stools of children with diarrhea (7,16).

Diarrhea duration at enrollment was categorized as 0-6 days of diarrhea versus prolonged diarrhea of 7-14 days, and diarrhea frequency in the past 24 hours was categorized as low frequency (3-6 stools) versus high frequency (>6 stools). Bivariate(unadjusted) and multivariable analyses were conducted. Multivariable models included covariates for potential confounding factors that may be associated with both child nutritional status and diarrhea etiology based on a literature review. All

Confidential: For Review Only

statistical analyses were performed using R version 4.0.2 Software (R Foundation for Statistical Computing).

RESULTS

The parent ABCD trial enrolled 8,268 participants who presented with MSD. In total, 6,699 had qPCR testing on stool samples collected at baseline, prior to any intervention. Of those, 2 had missing/invalid qPCR results and 5 had no bacterial diarrhea etiology. Upon recalculation of anthropometric indexes, seven participants were deemed to have been erroneously enrolled, as they did not meet all enrolment criteria. The deviations from anthropometric enrolment cut-offs were, however, minor and these seven children were retained in the sample. Therefore, 6,692 participants across the study sites were included in this analysis (Figure 1) and 28% overall had diarrhea of likely bacterial etiology (Table 1).

We first examined the association of MSD-defining characteristics with likely bacterial etiology. Compared to children with severe stunting and adjusting for multiple clinical and demographic factors, children presenting with some or severe dehydration only (adjusted odds ratio 1.67 [95% confidence intervals [1.26, 2.24]), MAM (aOR 1.57 [1.19,2.09]), and both MAM and dehydration (aOR 2.23 [1.62,3.08]) were significantly at higher odds of a likely bacterial infection (Table 2). A similar trend was noted for ST-ETEC etiology where children presenting with dehydration (aOR 1.74 [1.19,2.62]), MAM (aOR 1.69 [1.15,2.53]), and MAM and dehydration (aOR 1.83 [1.19,2.89]) compared to those severely stunted, were at higher odds of a likely ST-ETEC etiology. For *Shigella*, only children presenting with MAM and dehydration were found to have higher odds (aOR 1.64 [1.09,2.50]) compared to those severely stunted (Table 2). In bivariate associations of likely bacterial etiology, MAM and dehydration increased odds of likely bacterial etiology (OR 1.70 [1.28,2.27]) and MAM increased the odds of likely ST-ETEC etiology (OR 1.58 [1.13,2.26]) versus severe stunting (Supplementary table 1).

Confidential: For Review Only

Fever and duration of diarrhea were not associated with bacterial etiology or *Shigella* or ST-ETEC etiology (Table 2). However, children with high stool frequency (>6 stools) had increased odds of likely bacterial etiology (aOR 1.20 [1.05,1.36]) compared to those with lower stool frequency.

Since the qPCR analysis often detected more than one type of enteropathogen, we performed sensitivity analyses to evaluate the association between clinical and anthropometric characteristics with likely bacterial diarrhea etiology in children where only the enteropathogen of interest was found. The proportion of children who had only a single likely diarrhea bacterial etiology was 1,110/6,648(16.7%). No statistically significant associations were observed between anthropometrics and specific likely bacterial pathogens in these sensitivity analyses. High stool frequency, however, was associated with higher odds of likely bacterial diarrhea etiology (aOR 1.23 [1.05,1.45]), and *Shigella* etiology (aOR 1.32 [1.04,1.68]) after adjusting for multiple factors (Supplementary Tables 2 and 3).

DISCUSSION

In this analysis of 6,692 children with MSD whose etiology was assessed by qPCR methods, we found that several anthropometric and clinical factors were associated with likely bacterial diarrhea etiology. Children presenting with either MAM, dehydration or a combination of both, had higher odds of a likely bacterial and ST-ETEC diarrhea etiology compared to severely stunted children. Children with MAM and dehydration were also more likely to have *Shigella* diarrhea etiology compared to severely stunted children. Children with high stool frequency had higher odds of likely bacterial diarrhea etiology, a finding confirmed on sensitivity analysis evaluating children with only a single pathogen identified.

Our findings of association between MAM and a likely bacterial and ST-ETEC diarrhea etiology are consistent with some earlier studies (7,17). Tickell *et al*. studied 1,363 Kenyan children 6-59 months old who presented with acute diarrhea. The study noted that children with acute diarrhea and wasting (defined as MUAC <12.5 cm) were 1.8 times more likely to have enteroaggregative *E. coli* diarrhea etiology

https://mc.manuscriptcentral.com/bmjpo

Confidential: For Review Only

compared to those not wasted. Although the definition of acute malnutrition was different from our definition, they used similar PCR-based automated bacterial pathogen detection methods. A case-control study among 6-23 month old children in Bangladesh(17) noted that bacterial enteropathogens such as *Shigella* and EAEC were more common among children who were underweight (defined as WAZ<-2), a finding similar to ours. This study, however, enrolled children with malnutrition and only ~5% of those enrolled had diarrhea at baseline, whereas in our study all children had MSD. Similarly, a recent cross-sectional study carried out in Ethiopia noted that being wasted was associated with Salmonella infection (18).

Children with some/severe dehydration, with/without MAM, had greater odds of likely bacterial or ST-ETEC diarrhea etiology compared to children with severe stunting. Some/severe dehydration is known to be a risk factor for severe diarrhea (and even death) in children but not usually associated with bacterial diarrhea (19,20). In a study carried out in chidren <5 years presenting to a health clinic in Rwanda, *Shigella* was not associated with severe dehydration(21). Moreover, in a retrospective study carried out in Lao PDR to evaluate clinical and etiology associated with severity of acute diarrhea, among hospitalized children, 48.4% presented with dehydration and prevalence of dehydration was higher in those with rotavirus etiology versus rotavirus negative children. This association of dehydration was not noted in children with *Shigella* or *Salmonella* etiology, although the low numbers of samples positive for *Salmonella*(n=4) and *Shigella*(n=1) versus rotavirus (n=10) is noteworthy (22). Neither of the studies noted the association of dehydration in any bacterial diarrhea in contrast with our findings.

A re-analysis of the ABCD trial including molecular diagnostics was conducted to understand the effect of azithromycin in the outcomes in children with and without bacterial diarrhea etiology (15). It was noted that among those with likely and possible bacterial etiology, those in the azithromycin arm had lower risk of diarrhea at 3-days after randomization in comparison to those in the placebo arm.

Confidential: For Review Only

Additionally, within the 90-day follow-up period, azithromycin use was associated with lower risk of deaths/hospitalizations in children with likely and possible bacterial diarrhea. As noted in Pavlinac et al.'s results and previous studies, bacterial diarrhea may not always present with clinical signs and symptoms of dysentery (12,23) and therefore, limiting antibiotics to those with clinically apparent dysentery may limit treatment of bacterial diarrhea. The findings from our analysis outlines clinical and nutritional risk factors to potentially identify such high risk children who may benefit from antibiotics before culture results are known.

Our secondary analysis has some limitations. First, the observational, cross-sectional nature of the analysis limits the ability to make causal inferences. Second, we may not have had adequate power to detect more moderate magnitudes of association between nutritional status and diarrhea etiology. Third, our study included children with MSD which may limit the generalizability of our findings. Finally, the use of enteropathogens cut-offs is complicated in settings where asymptomatic carriage of enteropathogens is high. It is noteworthy that these cut-offs were derived from previous case-control studies, thus may be subject to further refinement. It is also noted that there were a small number of children with individual pathogens which limited the power to detect differences between groups. Strengths of the study include the multi-country setting, the prospective nature of the data collection, the inclusion of numerous possible confounding variables, standardized measures of anthropometric data, and the use of novel enteropathogen detection tools. We also note that additional co-variates that could influence the associations such as pre-term birth, bloody diarrhea, severe wasting were not included since some were not measured and others were exclusion criteria..

In summary, in this large cohort of children with MSD, we note that moderate acute malnutrition and dehydration as well as high frequency of stools were associated with an increased odds of likely bacterial etiology, compared to those with severe stunting. These findings suggest that these

Confidential: For Review Only

characteristics could be helpful criteria for children presenting with moderate to severe diarrhea who might benefit from antibiotics during an acute diarrheal episode. Future studies could examine other, more specific risk factors for diarrhea etiology, including micronutrient status (24,25), exposure to food and water sources (26–28), vaccine status (29) and others. Additional trials of the utility of targeted antibiotic therapy also seem warranted.

Acknowledgements: We would like to thank the families and children who participated in this trial as well as the staff who helped carried out the trial in each of the 7 sites. We also thank additional members of the ABCD Study Group: Bangladesh: Muhammad Waliur Rahman, Irin Parvin, and Md. Farhad Kabir. India: Pratibha Dhingra, Arup Dutta, Anil Kumar Sharma, Vijay Kumar Jaiswal. Kenya: Churchil Nyabinda, Christine McGrath, Emily L. Deichsel, Maurine Anyango, Kevin Mwangi Kariuki, Doreen Rwigi, and Stephanie N. Tornberg-Belanger. Mali: Fadima Cheick Haidara, Flanon Coulibaly, Jasnehta Permala-Booth, and Dramane Malle. Malawi: Nigel Cunliffe, Latif Ndeketa, Desiree Witte, Chifundo Ndamala. Pakistan: Shahida Qureshi, Sadia Shakoor, Rozina Thobani, and Jan Mohammed. Tanzania: Rodrick Kisenge, Mohamed Bakari, Cecylia Msemwa, and Abraham Samma. TAC: James Platts-Mills and Jie Liu.

Contributors: SS, PA, KM, CD, CS: Conceptualization. All authors: Approval and Validation of concept. SS, PA, KP, CD, CS: Methodology. SS, CR – Analysis. SS, CD: Writing – original draft. All authors: Critical review, comments and edits of paper. PA, KM, CD: Supervision

Funding: This trial was funded by the Bill & Melinda Gates Foundation (grant OPP 1126331). Under the grant conditions of the Foundation, a Creative Commons Attribution 4.0 Generic License has already been assigned to the Author Accepted Manuscript version that might arise from this submission. The study sponsor had no role in the analysis, interpretation of results, writing of this article or decision to submit

Confidential: For Review Only

this paper for publication. CPD was supported, in part, by the National Institutes of Health (K24 DK104676 and P30 DK040561)

Competing interests: None declared

Patient Consent for publication: Not applicable

Ethics approval: The ABCD trial (ClinicalTrials.gov Identifier: NCT03130114) was approved by the WHO Ethics Review Committee and the institutional review boards of all participating sites; Bangladesh (International Centre for Diarrhoeal Disease Research), India (Institutional Ethics Committee, Subharti Medical College & Hospital, Swami Vivekan and Subharti University), Kenya (Kenya Medical Research Institute Ethical Review Committee and the University of Washington Institutional Review Board), Malawi (University of Malawi College of Medicine Research Ethics Committee), Mali (Comite d' Ethiquedela FMPOS (ERCatUSTTB)), Pakistan (Aga Khan University, Ethical Review Committee), Tanzania (Muhimbili University of Health and Allied Sciences Senate Research and Publications Committee; Tanzanian Food and Drug Administration; National Institute for Medical Research), and United States (Boston Children's Hospital Institutional Review Board; University of Maryland, Baltimore Research Ethics Committee).

Data availability statement: The de-identified patient data are available upon reasonable request.

Confidential: For Review Only

Confidential: For Review Only

REFERENCES:

1. Troeger C, Blacker BF, Khalil IA, Rao PC, Cao S, Zimsen SR, et al. Estimates of the global, regional, and national morbidity, mortality, and aetiologies of diarrhoea in 195 countries: a systematic analysis for the Global Burden of Disease Study 2016. Lancet Infect Dis. 2018 Nov 1;18(11):1211–28.

2. Nuzhat S, Shahunja KM, Shahid ASMSB, Khan SH, Islam SB, Islam MR, et al. Diarrhoeal children with concurrent severe wasting and stunting compared to severe wasting or severe stunting. Trop Med Int Health. 2020;25(8):928–35.

3. Liu J, Platts-Mills JA, Juma J, Kabir F, Nkeze J, Okoi C, et al. Use of quantitative molecular diagnostic methods to identify causes of diarrhoea in children: a reanalysis of the GEMS case-control study. Lancet Lond Engl. 2016 Sep 24;388(10051):1291–301.

4. World Health Organization, Department of Child and Adolescent Health and Development. The treatment of diarrhoea: a manual for physicians and other senior health workers. Geneva: Dept. of Child and Adolescent Health and Development, World Health Organization; 2005.

5. Mondal D, Haque R, Sack RB, Kirkpatrick BD, Petri WA. Attribution of Malnutrition to Cause-Specific Diarrheal Illness: Evidence from a Prospective Study of Preschool Children in Mirpur, Dhaka, Bangladesh. Am J Trop Med Hyg. 2009 May;80(5):824–6.

6. Ferdous F, Das SK, Ahmed S, Farzana FD, Latham JR, Chisti MJ, et al. Severity of Diarrhea and Malnutrition among Under Five-Year-Old Children in Rural Bangladesh. Am J Trop Med Hyg. 2013 Aug 7;89(2):223–8.

7. Tickell KD, Pavlinac PB, John-Stewart GC, Denno DM, Richardson BA, Naulikha JM, et al. Impact of Childhood Nutritional Status on Pathogen Prevalence and Severity of Acute Diarrhea. Am J Trop Med Hyg. 2017 Nov;97(5):1337–44.

8. ABCD study team. A double-blind placebo-controlled trial of azithromycin to reduce mortality and improve growth in high-risk young children with non-bloody diarrhoea in low resource settings: the Antibiotics for Children with Diarrhoea (ABCD) trial protocol. Trials. 2020 Jan 13;21(1):71.

9. The Antibiotics for Children With Diarrhea (ABCD) Study Group. Effect of 3 Days of Oral Azithromycin on Young Children With Acute Diarrhea in Low-Resource Settings: A Randomized Clinical Trial. JAMA Netw Open. 2021 Dec 16;4(12):e2136726.

10. Colston JM, Ahmed AMS, Soofi SB, Svensen E, Haque R, Shrestha J, et al. Seasonality and within-subject clustering of rotavirus infections in an eight-site birth cohort study. Epidemiol Infect. 2018 Apr;146(6):688–97.

11. Pavlinac PB, Platts-Mills J, Liu J, Atlas HE, Gratz J, Operario DJ, et al. Effect of azithromycin on bacterial acute watery diarrhea: A re-analysis of the AntiBiotics for Children with severe Diarrhea (ABCD) trial incorporating molecular diagnostics. J Infect Dis.

12. Platts-Mills JA, Liu J, Rogawski ET, Kabir F, Lertsethtakarn P, Siguas M, et al. Use of quantitative molecular diagnostic methods to assess the aetiology, burden, and clinical characteristics of diarrhoea in children in low-resource settings: a reanalysis of the MAL-ED cohort study. Lancet Glob Health. 2018 Dec;6(12):e1309–18.

13. Kotloff KL, Blackwelder WC, Nasrin D, Nataro JP, Farag TH, van Eijk A, et al. The Global Enteric Multicenter Study (GEMS) of Diarrheal Disease in Infants and Young Children in Developing Countries: Epidemiologic and Clinical Methods of the Case/Control Study. Clin Infect Dis Off Publ Infect Dis Soc Am. 2012 Dec 15;55(Suppl 4):S232–45.

14. The MAL-ED Network Investigators. The MAL-ED Study: A Multinational and Multidisciplinary Approach to Understand the Relationship Between Enteric Pathogens, Malnutrition, Gut Physiology, Physical Growth, Cognitive Development, and Immune Responses in Infants and Children Up to 2 Years of Age in Resource-Poor Environments. Clin Infect Dis. 2014 Nov 1;59(suppl_4):S193–206.

15. Pavlinac PB, Platts-Mills JA, Liu J, Atlas HE, Gratz J, Operario D, et al. Azithromycin for Bacterial Watery Diarrhea: A Reanalysis of the AntiBiotics for Children With Severe Diarrhea (ABCD) Trial Incorporating Molecular Diagnostics. J Infect Dis. 2023 Jul 5;jiad252.

16. Kotloff KL, Nataro JP, Blackwelder WC, Nasrin D, Farag TH, Panchalingam S, et al. Burden and aetiology of diarrhoeal disease in infants and young children in developing countries (the Global Enteric Multicenter Study, GEMS): a prospective, case-control study. The Lancet. 2013 Jul 20;382(9888):209–22.

17. Platts-Mills JA, Taniuchi M, Uddin MJ, Sobuz SU, Mahfuz M, Gaffar SA, et al. Association between enteropathogens and malnutrition in children aged 6–23 mo in Bangladesh: a case-control study. Am J Clin Nutr. 2017 May 1;105(5):1132–8.

18. Balew M, Kibret M. Prevalence of enteric bacterial pathogens in diarrheic under-five children and their association with the nutritional status in Bahir Dar Zuria District, Northwest Ethiopia. BMC Nutr. 2023 Feb 24;9(1):35.

19. Griffin PM, Ryan CA, Nyaphisi M, Hargrett-Bean N, Waldman RJ, Blake PA. Risk factors for fatal diarrhea: a case-control study of African children. Am J Epidemiol. 1988 Dec;128(6):1322–9.

20. O'Reilly CE, Jaron P, Ochieng B, Nyaguara A, Tate JE, Parsons MB, et al. Risk factors for death among children less than 5 years old hospitalized with diarrhea in rural western Kenya, 2005-2007: a cohort study. PLoS Med. 2012;9(7):e1001256.

21. Kabayiza JC, Andersson ME, Nilsson S, Baribwira C, Muhirwa G, Bergström T, et al. Diarrhoeagenic microbes by real-time PCR in Rwandan children under 5 years of age with acute gastroenteritis. Clin Microbiol Infect. 2014 Dec 1;20(12):O1128–35.

Confidential: For Review Only

22. Vorlasane L, Luu MN, Tiwari R, Imoto A, Sato M, Huy NT, et al. The clinical characteristics, etiologic pathogens and the risk factors associated with dehydration status among under-five children hospitalized with acute diarrhea in Savannakhet Province, Lao PDR. PLOS ONE. 2023 Mar 2;18(3):e0281650.

23. Tickell KD, Brander RL, Atlas HE, Pernica JM, Walson JL, Pavlinac PB. Identification and management of Shigella infection in children with diarrhoea: a systematic review and meta-analysis. Lancet Glob Health. 2017 Dec 1;5(12):e1235–48.

24. Khalili B, Hart A, Mardani M, Khalili M, McArdle F, Cuevas L. Diarrhea-associated micronutrient deficiencies and risk of subsequent diarrhea in admitted children to Hajar hospital in Shahrekord, Iran. Arch Clin Infect Dis [Internet]. 2007 [cited 2023 Mar 7]; Available from: https://www.semanticscholar.org/paper/Diarrhea-associated-micronutrient-deficiencies-and-Khalili-Hart/452eb0a4188665e4003d25c2dab055720b2262e4

25. Talachian E, Bidari A, Noorbakhsh S, Tabatabaei A, Salari F. Serum levels of vitamins A and D, and zinc in children with acute diarrhea: A cross-sectional study. Med J Islam Repub Iran. 2015 Apr 27;29:207.

26. George CM, Perin J, Neiswender de Calani KJ, Norman WR, Perry H, Davis TP, et al. Risk Factors for Diarrhea in Children under Five Years of Age Residing in Peri-urban Communities in Cochabamba, Bolivia. Am J Trop Med Hyg. 2014 Dec 3;91(6):1190–6.

27. Maponga BA, Chirundu D, Gombe NT, Tshimanga M, Shambira G, Takundwa L. Risk factors for contracting watery diarrhoea in Kadoma City, Zimbabwe, 2011: a case control study. BMC Infect Dis. 2013 Dec 2;13(1):567.

28. Knight SM, Toodayan W, Caique WC, Kyi W, Barnes A, Desmarchelier P. Risk Factors for the Transmission of Diarrhoea in Children: A Case-Control Study in Rural Malaysia. Int J Epidemiol. 1992 Aug 1;21(4):812–8.

29. Bahartha AS, AlEzzi JI. Risk factors of diarrhea in children under 5 years in Al-Mukalla, Yemen. Saudi Med J. 2015 Jun;36(6):720–4.

Confidential: For Review Only

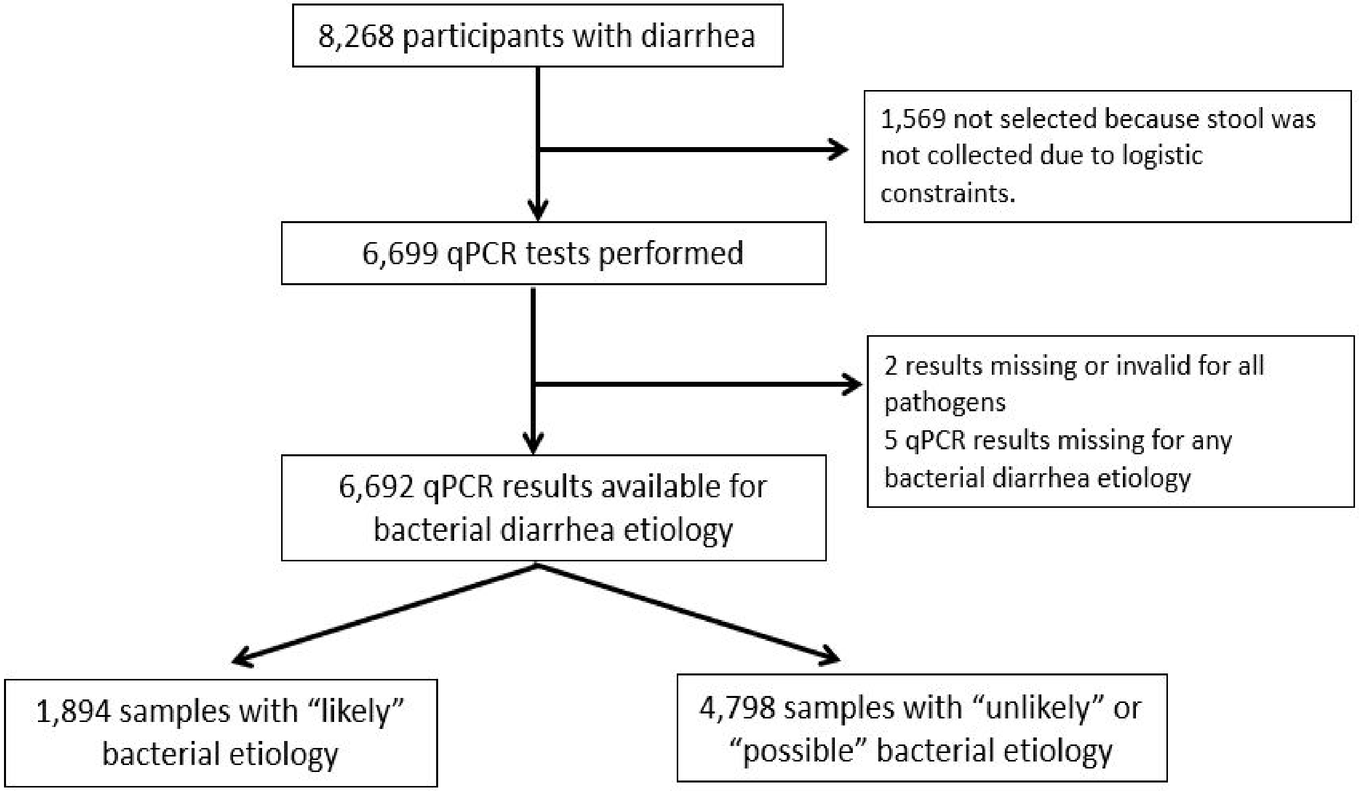

Figure 1: Flow chart of 2–23-month-old children presenting with moderate to severe diarrhea.

"Likely" diarrhea associated etiology was determined by Ct value cut-offs for specific enteropathogens. Ct values greater than these cut-offs but < 35 were considered "possible" etiologies while Ct value >35 was considered "Unlikely" etiology

Table 1: Baseline characteristics of 6,692 mothers and their 2-23-month-old children who presented with moderate-to-severe diarrhea.

**Baseline characteristics**	**Bangladesh (n=1000)**	**India (n=998)**	**Pakistan (n=997)**	**Kenya (n=1014)**	**Malawi (n=691)**	**Mali (n=1000)**	**Tanzania (n=997)**	**ALL SITES (n=6,692)**
Age (months)	11.2 (5.0)[*]	11.9 (5.6)[*]	12.5 (5.5)[*]	11.0 (5.7)[*]	12.0 (4.8)[*]	12.0 (4.6)[*]	11.0 (5.2)[*]	11.6 (5.3)[*]
Males	57.8%	52.9%	50.6%	52.7%	56.9%	53.9%	53.2%	53.8%
Any rotavirus vaccination	1.0%	2.7%	7.3%	99.6%	99.6%	97.6%	99.9%	60%
Exclusively breastfed at time of enrolment	1.7%	6.9%	9.2%	17.9%	4.1%	5.4%	14.4%	8.7%
Maternal age, years	24 (5)	26 (4)	27 (6)	26 (6)	25 (6)	26 (6)	27 (6)	26 (6)
Maternal body mass index, kg/m2	22.3 (4.3)	22.6 (4.3)	23.2 (5.0)	23.2 (3.8)	23.1 (5.3)	24.0 (4.9)	24.5 (5.0)	23.3 (4.7)
Maternal education, completed school years	5.8 (4.0)	4.8 (4.8)	2.9 (4.0)	9.0 (2.9)	8.3 (2.8)	4.3 (5.0)	7.8 (2.7)	6.0 (4.4)
Number of children <5 years of age in house	1.2 (0.4)	1.9 (0.9)	2.0 (1.0)	1.7 (0.8)	1.4 (0.6)	2.4 (1.8)	1.3 (0.5)	1.7 (1.1#)
Fever (defined as temperature >37.5ºC)	15.3%	7.6%	9.0%	6.0%	19.4%	16.9%	12.5%	12.1%
Some/severe dehydration	25.1%	48.4%	28.5%	91.8%	77.7%	23.1%	92.8%	54.4%
Length for age z-score (LAZ)	-1.83 (1.15)	-2.12 (1.28)	-2.15 (1.28)	-0.81 (1.26)	-1.55 (1.47)	-1.16 (1.13)	-0.76 (1.14)	-1.48 (1.36)
Weight for length z-score (WLZ)	-1.69 (0.90)	-1.37 (1.01)	-1.45 (0.96)	-0.28 (1.19)	-0.62 (1.19)	-2.04 (0.74)	-0.39 (1.27)	-1.14 (1.23)
Severe stunting[†]	19.5%	27.6%	27.5%	5.6%	16.5%	6.5%	2.2%	15.0%
Moderate Acute Malnutrition[‡]	73.6%	57.3%	70.6%	14.0%	26.0%	85.0%	16.1%	49.9%
Presence[§] of any bacterial pathogen[**] in stool	36.3%	27.5%	30.9%	21.5%	31.7%	28.6%	22.9%	28.3%
Presence[5] of Shigella in stool	12.8%	16.0%	18.2%	7.0%	11.3%	13.5%	9.2%	12.6%
Presence[5] of ST-ETEC[††] in stool	19.4%	9.9%	12.7%	10.4%	20.4%	11.9%	10.7%	13.3%

[*] Numbers indicate percentages for categorical variable and mean (SD) for continuous variables
[†] Defined as Length for age z-score of < -3.0
[‡] Defined as Weight for Length z-score < -2.0 & ≥-3.00 or Mid-upper arm Circumference of <12.5 & ≥ 11.5 if participant was 6 months or older
[§] Indicated as a Cq value below relevant cut-off in a quantitative polymerase chain reaction (Liu et at., 2016).
[**] Includes the following bacteria - Cq cut-off for diagnostic quantity to be termed as associated with diarrhea: Campylobacter spp (16.3), typical EPEC (18.1), ST-ETEC (25.4), Salmonella spp (31.9), Shigella spp (28.7), Vibrio cholerae (32.6)
[††] (Heat stable Toxin) STh-producing enterotoxigenic E. Coli

Confidential: For Review Only

Table 2: Clinical and nutritional correlates of likely bacterial etiology of diarrhea among 2-23-month-old children with moderate-to-severe diarrhea adjusted for factors

	Bacterial etiology			*Escherichia coli* encoding heat-stable toxin etiology			*Shigella* etiology		
	**Proportion n/N (%)**	**Adjusted[*] odds ratio (95% CI)**	**p-value**	**Proportion n/N (%)**	**Adjusted[1] odds ratio (95% CI)**	**p-value**	**Proportion n/N (%)**	**Adjusted[1] odds ratio (95% CI)**	**p-value**
**Moderate to severe diarrhea defining characteristics**									
**Severe stunting only**	94/417 (22.5%)	Ref.	-	40/417 (9.6%)	Ref.	-	52/417 (12.5%)	Ref.	-
**Some /severe dehydration only**	764/2,832 (27.0%)	1.67 (1.26, 2.24)	<0.001	378/2815 (13.4%)	1.74 (1.19, 2.62)	0.006	305/2832 (10.8%)	1.18 (0.82, 1.73)	0.38
**MAM only**	675/2,228 (30.3%)	1.57 (1.19, 2.09)	0.002	319/ 2,227 (14.3%)	1.69 (1.15, 2.53)	0.009	320/2224 (14.4%)	1.29 (0.90, 1.86)	0.17
**MAM and some /severe dehydration**	209/626 (33.4%)	2.23 (1.62, 3.08)	<0.001	81/623 (13.0%)	1.83 (1.18, 2.89)	0.008	90/626 (14.4%)	1.64 (1.09, 2.50)	0.02
**MAM and severe stunting**	109/408 (26.7%)	1.13 (0.79, 1.62)	0.52	45/408 (11.0%)	1.04 (0.62,1.74)	0.87	63/408 (15.4%)	1.08 (0.69, 1.70)	0.75
**Some/severe dehydration and severe stunting**	22/95 (23.2%)	1.42 (0.79, 2.49)	0.23	14/95 (14.7%)	1.92 (0.92, 3.82)	0.07	5/95 (5.3%)	0.52 (0.17, 1.27)	0.19
**MAM, some/severe dehydration, and severe stunting**	20/84 (23.8%)	0.80 (0.39, 1.53)	0.52	12/84 (14.3%)	1.19 (0.46, 2.71)	0.69	10/84 (11.9%)	0.68 (0.25, 1.58)	0.41
**Fever**									
**No**	1,646/5,884 (28.0%)	Ref.	-	767/5,865 (13.1%)	Ref	-	736/5,883 (12.5%)	Ref	-
**Yes**	248/808 (30.7%)	1.11 (0.93, 1.32)	0.26	122/806 (15.1%)	1.12 (0.89, 1.40)	0.31	109/808 (13.5%)	1.15 (0.90, 1.46)	0.26
**Duration of diarrhea (excluding day of enrollment)**									
**Diarrhea (0-6 days)**	1,781/6,328 (28.1%)	Ref.	-	846/6,308 (13.4%)	Ref	-	783/6,327 (12.4%)	Ref	-
**Prolonged duration (7-14 days)**	113/364 (31.0%)	1.22 (0.94, 1.58)	0.13	43/363 (11.8%)	1.05 (0.72, 1.50)	0.79	62/364 (17.0%)	1.29 (0.92, 1.78)	0.13
**Frequency of diarrhea in the past 24 hours**									
**Low frequency (3-6 stools)**	912/3,536 (25.8%)	Ref.	-	433/3,521 (12.3%)	Ref	-	406/3,535 (11.5%)	Ref	-
**High frequency (>6 loose stools)**	982/3,156 (31.1%)	1.20 (1.05, 1.36)	0.007	456/3,150 (14.5%)	1.10 (0.93, 1.31)	0.28	439/3,156 (13.9%)	1.17 (0.98, 1.40)	0.08

Adjusted for: child age, child sex, household wealth quintile, maternal age, maternal education, country of enrolment, rotavirus vaccination status, maternal BMI, total number of children under age 5 year in the household, defining characteristic of moderate to severe diarrhea, fever, duration of diarrhea, and high vs. low frequency of stools in past 24h.

Confidential: For Review Only

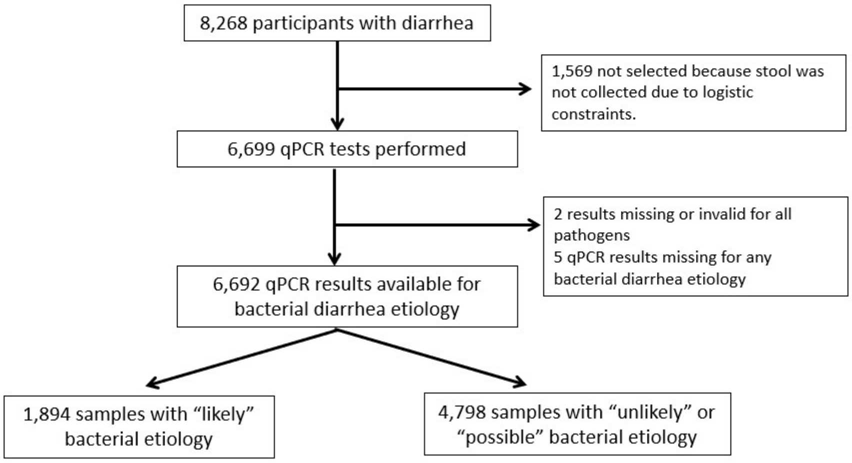

Figure 1: Flow chart of 2–23-month-old children presenting with moderate to severe diarrhea. “Likely” diarrhea associated etiology was determined by Ct value cut-offs for specific enteropathogens. Ct values greater than these cut-offs but < 35 were considered “possible” etiologies while Ct value >35 was considered “Unlikely” etiology.

176x100mm (144 x 144 DPI)

Supplementary tables

Table 1: Clinical and nutritional unadjusted odds ratios of likely bacterial etiology of diarrhea among 2-23-month-old children with moderate-to-severe diarrhea

	Bacterial etiology			Escherichia coli encoding heat-stable toxin etiology			Shigella etiology		
**Variable**	**Proportion n/N (%)**	**Unadjusted odds ratio (95% CI)**	**p-value**	**Proportion n/N (%)**	**Unadjusted odds ratio (95% CI)**	**p-value**	**Proportion n/N (%)**	**Unadjusted odds ratio (95% CI)**	**p-value**
**Moderate to severe diarrhea defining characteristics**									
**Severe stunting only**	94/417 (22.5%)	Ref	-	40/417 (9.6%)	Ref	-	52/417 (12.5%)	Ref	-
**Some / severe dehydration only**	764/2,832 (27.0%)	1.27 (1.00,1.63)	0.06	378/2815 (13.4%)	1.46 (1.05,2.09)	0.030	305/2832 (10.8%)	0.85 (0.62, 1.17)	0.30
**MAM only**	675/2,228 (30.3%)	1.47 (1.16,1.90)	0.93	319/ 2,227 (14.3%)	1.58 (1.13,2.26)	0.01	320/2224 (14.4%)	1.18 (0.87,1.63)	0.31
**MAM and some /severe dehydration**	209/626 (33.4%)	1.70 (1.28,2.27)	0.002	81/623 (13.0%)	1.41 (0.95,2.12)	0.09	90/626 (14.4%)	1.18 (0.82,1.71)	0.38
**MAM and severe stunting**	109/408 (26.7%)	1.24 (0.90,1.70)	0.19	45/408 (11.0%)	1.17 (0.75,1.84)	0.50	63/408 (15.4%)	1.28 (0.86,1.91)	0.22
**Some/severe dehydration and severe stunting**	22/95 (23.2%)	1.02 (0.59, 1.71)	0.93	14/95 (14.7%)	1.63 (0.82,3.07)	0.15	5/95 (5.3%)	0.39 (0.13,0.92)	0.051
**MAM, some/severe dehydration and severe stunting**	20/84 (23.8%)	1.06 (0.60,1.81)	0.84	12/84 (14.3%)	1.57 (0.76,3.06)	0.20	10/84 (11.9%)	0.95 (0.44, 1.88)	0.89
**Fever**									
**No**	1,646/5,884 (28.0%)	Ref	-	767/5,865 (13.1%)	Ref	-	736/5,883 (12.5%)	Ref	-
**Yes**	248/808 (30.7%)	1.14 (0.97,1.34)	0.11	122/806 (15.1%)	1.19 (0.96. 1.45)	0.11	109/808 (13.5%)	1.09 (0.88, 1.35)	0.43
**Duration of diarrhea (excluding day of enrollment)**									
**Diarrhea (0-6 days)**	1,781/6,328 (28.1%)	Ref	-	846/6,308 (13.4%)	Ref	-	783/6,327 (12.4%)	Ref	-
**Prolonged duration (7-14 days)**	113/364 (31.0%)	1.15 (0.91,1.44)	0.23	43/363 (11.8%)	0.87 (0.62, 1.19)	0.39	62/364 (17.0%)	1.45 (1.09,1.92)	0.010
**Frequency of diarrhea in the past 24 hours**									
**Low frequency (3-6 stools)**	912/3,536 (25.8%)	Ref	-	433/3,521 (12.3%)	Ref	-	406/3,535 (11.5%)	Ref	-
**High frequency (>6 loose stools)**	982/3,156 (31.1%)	1.30 (1.17, 1.45)	<0.001	456/3,150 (14.5%)	1.21 (1.05, 1.39)	0.009	439/3,156 (13.9%)	1.25 (1.08,1.44)	0.003

Confidential: For Review Only

Table 2: Clinical and nutritional correlates with likely bacterial diarrheal etiology with no coinfection including enterotoxigenic Escherichia coli (E.coli) encoding heat-stable toxin (ST-ETEC) and Shigella diarrheal etiology with no coinfection in 2-23-month-old children with moderate-to-severe diarrhea adjusted for factors.

	**Bacterial etiology**			***Escherichia coli* encoding heat-stable toxin etiology**			***Shigella* etiology**		
	**Proportion n/N (%)**	**Adjusted[1] odds ratio (95% CI)**	**p-value**	**Proportion n/N (%)**	**Adjusted[1] odds ratio (95% CI)**	**p-value**	**Proportion n/N (%)**	**Adjusted[1] odds ratio (95% CI)**	**p-value**
**Moderate to severe diarrhea defining characteristics**									
**Severe stunting only**	65/417 (15.6%)	Ref.	-	18/417 (4.3%)	Ref.	-	35/417 (8.4%)	Ref.	-
**Some /severe dehydration only**	464/2,336 (16.6%)	1.34 (0.96, 1.88)	0.088	156/2,824 (5.5%)	1.36 (0.79, 2.52)	0.30	150/2,829 (5.3%)	0.66 (0.42, 1.05)	0.074
**MAM only**	382/2,2224 (17.2%)	1.18 (0.86,1.65)	0.31	116/2,221 (5.2%)	1.28 (0.74, 2.37)	0.40	154/2,223 (6.9%)	0.95 (0.61, 1.50)	0.81
**MAM and some /severe dehydration**	107/623 (17.2%)	1.38 (0.94, 2.02)	0.10	28/625 (4.5%)	1.23 (0.63, 2.47)	0.55	30/626 (4.8%)	0.64 (0.36, 1.14)	0.13
**MAM and severe stunting**	71/407 (17.4%)	0.91 (0.59, 1.39)	0.66	20/407 (4.9%)	1.05 (0.50, 2.22)	0.89	33/407 (8.1%)	0.61 (0.33, 1.11)	0.11
**Some/severe dehydration and severe stunting**	8/95 (8.4%)	0.70 (0.29, 1.47)	0.37	5/95 (5.3%)	1.38 (0.43, 3.75)	0.55	2/95 (2.1%)	0.28 (0.04, 0.98)	0.09
**MAM, some/severe dehydration, and severe stunting**	14/84 (16.7%)	0.75 (0.31, 1.60)	0.49	6/84 (7.1%)	1.40 (0.39, 4.05)	0.57	3/84 (3.6%)	0.29 (0.05, 1.01)	0.098
**Fever**									
**No**	986/5,845 (16.9%)	Ref.	-	312/5,870 (5.3%)	Ref	-	364/5,880 (6.2%)	Ref	-
**Yes**	124/803 (15.4%)	0.88 (0.70,1.10)	0.29	36/806 (4.5%)	0.79 (0.53, 1.15)	0.24	43/763 (5.3%)	0.88 (0.60,1.27)	0.52
**Duration of diarrhea (excluding day of enrollment)**									
**Diarrhea (0-6 days)**	1,036/ 6,285 (16.5%)	Ref.	-	329/6,314 (5.2%)	Ref	-	32/363 (8.8%)	Ref	-
**Prolonged duration (7-14 days)**	74/363 (20.4%)	1.22 (0.90,1.64)	0.19	19/362 (5.2%)	1.05 (0.60,1.73)	0.85	375/6,323 (5.9%)	1.19 (0.75,1.83)	0.44
**Frequency of diarrhea in the past 24 hours**									
**Low frequency (3-6 stools)**	534/3,508 (15.2%	Ref.	-	195/3,528 (5.5%)	Ref	-	202/3,534 (5.7%)	Ref	-
**High frequency (>6 loose stools)**	576/3,140 (18.3%)	1.23 (1.05,1.45)	0.0091	153/3,150 (4.9%)	0.99 (0.76, 1.28)	0.94	205/3,152 (6.5%)	1.32 (1.04,1.68)	0.025

Adjusted for: child age, child sex, household wealth quintile, maternal age, maternal education, country of enrolment, rotavirus vaccination status, maternal BMI, total number of children under age 5 year in the household, defining characteristic of moderate to severe diarrhea, fever, duration of diarrhea, and high vs. low frequency of stools in past 24h.

Confidential: For Review Only

Table 3: Clinical and nutritional correlates with bacterial diarrheal etiology with no coinfection including enterotoxigenic Escherichia coli (E.coli) encoding heat-stable toxin (ST-ETEC) and Shigella diarrheal etiology with no coinfection in 2-23-month-old children with moderate-to-severe diarrhea.

	Bacterial etiology			Escherichia coli encoding heat-stable toxin etiology			Shigella etiology		
**Variable**	**Proportion n/N (%)**	**Unadjusted odds ratio (95% CI)**	**p-value**	**Proportion n/N (%)**	**Unadjusted odds ratio (95% CI)**	**p-value**	**Proportion n/N (%)**	**Unadjusted odds ratio (95% CI)**	**p-value**
**Moderate to severe diarrhea defining characteristics**									
**Severe stunting only**	65/417 (15.6%)	Ref	-	18/417 (4.3%)	Ref.	-	35/417 (8.4%)	Ref	-
**Some / severe dehydration only**	464/2,336 (16.6%)	1.08 (0.82,1.44)	0.61	156/2,824 (5.5%)	1.30 (0.81,2.21)	0.31	150/2,829 (5.3%)	0.61 (0.42,0.91)	0.01
**MAM only**	382/2,2224 (17.2%)	1.12 (0.85, 1.51)	0.43	116/2,221 (5.2%)	1.22 (0.75,2.09)	0.45	154/2,223 (6.9%)	0.81 (0.56, 1.21)	0.28
**MAM and some /severe dehydration**	107/623 (17.2%)	1.12 (0.80,1.58)	0.50	28/625 (4.5%)	1.04 (0.57,1.94)	0.90	30/626 (4.8%)	0.55 (0.33,0.91)	0.020
**MAM and severe stunting**	71/407 (17.4%)	1.14 (0.79, 1.65)	0.48	20/407 (4.9%)	1.14 (0.59, 2.21)	0.69	33/407 (8.1%)	0.96 (0.58,1.58)	0.87
**Some/severe dehydration and severe stunting**	8/95 (8.4%)	0.50 (0.21, 1.02)	0.076	5/95 (5.3%)	1.23 (0.39, 3.18)	0.69	2/95 (2.1%)	0.24 (0.04, 0.79)	0.049
**MAM, some/severe dehydration and severe stunting**	14/84 (16.7%)	1.08 (0.56, 1.99)	0.80	6/84 (7.1%)	1.71 (0.60, 4.21)	0.27	3/84 (3.6%)	0.40 (0.09, 1.16)	0.14
**Fever**									
**No**	986/5,845 (16.9%)	Ref	-	312/5,870 (5.3%)	Ref	-	364/5,880 (6.2%)	Ref	-
**Yes**	124/803 (15.4%)	0.90 (0.73, 1.10)	0.31	36/806 (4.5%)	0.83 (0.58, 1.17)	0.31	43/763 (5.3%)	0.85 (0.61,1.17)	0.34
**Duration of diarrhea (excluding day of enrollment)**									
**Diarrhea (0-6 days)**	1,036/ 6,285 (16.5%)	Ref	-	329/6,314 (5.2%)	Ref	-	32/363 (8.8%)	Ref	-
**Prolonged duration (7-14 days)**	74/363 (20.4%)	1.30 (0.99,1.68)	0.05	19/362 (5.2%)	1.01 (0.61, 1.58)	0.98	375/6,323 (5.9%)	1.53 (1.03,2.20)	0.03
**Frequency of diarrhea in the past 24 hours**									
**Low frequency (3-6 stools)**	534/3,508 (15.2%	Ref	-	195/3,528 (5.5%)	Ref	-	202/3,534 (5.7%)	Ref	-
**High frequency (>6 loose stools)**	576/3,140 (18.3%)	1.25 (1.10,1.42)	<0.001	153/3,150 (4.9%)	0.87 (0.70, 1.08)	0.22	205/3,152 (6.5%)	1.15 (0.94, 1.40)	0.18

Confidential: For Review Only

BMJ Paediatrics Open

Clinical and nutritional correlates of bacterial diarrhea etiology in young children: A secondary cross-sectional analysis of the ABCD trial

Journal:	*BMJ Paediatrics Open*
Manuscript ID	bmjpo-2023-002448.R1
Article Type:	Original research
Date Submitted by the Author:	02-Mar-2024
Complete List of Authors:	Somji, Sarah; Tampere University, Center for Child, Adolescent, and Maternal Health Research, Faculty of Medicine and Health Technology; Muhimbili University of Health and Allied Sciences, Department of Epidemiology<br>Ashorn, Per; Tampere University, Center for Child, Adolescent, and Maternal Health Research, Faculty of Medicine and Health Technology<br>Manji, Karim; Muhimbili University of Health and Allied Sciences, Pediatrics<br>Ahmed, Tahmeed; International Centre for Diarrhoeal Disease Research Bangladesh, Nutrition and Clinical Sciences Division<br>Chisti, Md.; International Centre for Diarrhoeal Disease Research Bangladesh, Nutrition and Clinical Services Devision<br>Dhingra, Usha; Center for Public Health Kinetics<br>Sazawal, Sunil; Centre for Public Health Kinetics (CPHK)<br>Singa, Benson; Kenya Medical Research Institute, Center for Clinical Research<br>Walson, Judd L.; University of Washington, Department of Global Health; University of Washington, Department of Epidemiology<br>Pavlinac, Patricia; University of Washington, Department of Global Health; University of Washington, Department of Epidemiology<br>Bar-Zeev, Naor; Johns Hopkins Bloomberg School of Public Health, International Vaccine Access Center<br>Houpt, Eric; University of Virginia, Department of Medicine, Infectious Diseases<br>Dube, Queen; Queen Elizabeth Central Hospital, Department of Pediatrics<br>Kotloff, Karen; University of Maryland Baltimore, Center for Vaccine Development and Global Health; University of Maryland, Department of Pediatrics, Center for Vaccine Development and Global Health<br>Sow, Samba; Centre pour le Developpement des Vaccins Mali<br>Yousafzai, Mohammad Tahir; The Aga Khan University<br>Qamar, Farah; The Aga Khan University, Department of Pediatrics and Child Heath<br>Bahl, Rajiv ; World Health Organization, Department of Maternal, Child, and Adolescent Health and Aging<br>De Costa, Ayesha; World Health Organization, Department of Maternal, Child, and Adolescent Health and Aging<br>Simon, Jonathon; World Health Organization, Department of Maternal,

	Child, and Adolescent Health and Aging<br>Sudfeld, Christopher; Harvard University T H Chan School of Public Health, Department of Global Health and Population and Nutrition<br>Duggan, Christopher; Boston Children's Hospital, Division of Gastroenterology, Hepatology and Nutrition; Harvard University T H Chan School of Public Health, Department of Global Health and Population and Nutrition<br>Study Group, ABCD; ABCD Study Group
Keywords:	Epidemiology, Infant

SCHOLARONE™
Manuscripts

I, the Submitting Author has the right to grant and does grant on behalf of all authors of the Work (as defined in the below author licence), an exclusive licence and/or a non-exclusive licence for contributions from authors who are: i) UK Crown employees; ii) where BMJ has agreed a CC-BY licence shall apply, and/or iii) in accordance with the terms applicable for US Federal Government officers or employees acting as part of their official duties; on a worldwide, perpetual, irrevocable, royalty-free basis to BMJ Publishing Group Ltd ("BMJ") its licensees and where the relevant Journal is co-owned by BMJ to the co-owners of the Journal, to publish the Work in this journal and any other BMJ products and to exploit all rights, as set out in our licence.

The Submitting Author accepts and understands that any supply made under these terms is made by BMJ to the Submitting Author unless you are acting as an employee on behalf of your employer or a postgraduate student of an affiliated institution which is paying any applicable article publishing charge ("APC") for Open Access articles. Where the Submitting Author wishes to make the Work available on an Open Access basis (and intends to pay the relevant APC), the terms of reuse of such Open Access shall be governed by a Creative Commons licence – details of these licences and which Creative Commons licence will apply to this Work are set out in our licence referred to above.

Other than as permitted in any relevant BMJ Author's Self Archiving Policies, I confirm this Work has not been accepted for publication elsewhere, is not being considered for publication elsewhere and does not duplicate material already published. I confirm all authors consent to publication of this Work and authorise the granting of this licence.

Confidential: For Review Only

Clinical and nutritional correlates of bacterial diarrhea etiology in young children:

A secondary cross-sectional analysis of the ABCD trial

Sarah Somji[1,2], Per Ashorn[1], Karim Manji [3], Tahmeed Ahmed[4], MD. Chisti[4], Usha Dhingra[5], Sunil Sazawal[5], Benson Singa[6], Judd L Walson[7,8,9], Patricia Pavlinac[7,8], Naor Bar-Zeev[10], Eric Houpt[11], Queen Dube[12], Karen Kotloff[13,14], Samba Sow[15], Mohammad Tahir Yousafzai[16], Farah Qamar[16], Rajiv Bahl[17], Ayesha De Costa[17], Jonathon Simon[17], Christopher Sudfeld*[18], Christopher P. Duggan*[18,19] and the ABCD Study Group**

**contributed equally*

*** Listed in the acknowledgements*

Corresponding Author: Sarah Somji, PhD Student, Center for Child, Adolescent, and Maternal Health Research, Faculty of Medicine and Health Technology, Tampere University and Tampere University Hospital, Tampere, Finland; sarahsomji@gmail.com and Department of Epidemiology, Muhimbili University of Health and Allied Sciences, Dar-es-Salaam, Tanzania

Author affiliations:

1. Center for Child, Adolescent, and Maternal Health Research, Faculty of Medicine and Health Technology, Tampere University and Tampere University Hospital, Tampere, Finland
2. Department of Epidemiology, Muhimbili University of Health and Allied Sciences, Dar-es-Salaam, Tanzania

Confidential: For Review Only

3. Department of Pediatrics, Muhimbili University of Health and Allies Sciences, Dar-es-Salaam, Tanzania
4. Nutrition and Clinical Services Division, International Centre for Diarrheal Disease Research, Bangladesh, Dhaka, Bangladesh
5. Center for Public Health Kinetics (CPHK), New Delhi, India
6. Kenya Medical Research Institute (KEMRI), Center for Clinical Research, Nairobi, Kenya
7. Department of Global Health, University of Washington, Seattle USA
8. Department of Epidemiology, University of Washington, Seattle USA
9. Department of Pediatrics and Medicine (Infectious Diseases), University of Washington, Seattle, USA
10. International Vaccine Access Center, Johns Hopkins Bloomberg School of Public Health, Baltimore, Maryland
11. Department of Medicine, Infectious Diseases ; International Health, University of Virginia, Charlottesville, USA
12. Department of Pediatrics, Queen Elizabeth Central Hospital, Blantyre, Malawi
13. Center for Vaccine Development and Global Health, University of Maryland School of Medicine, Baltimore
14. Department of Pediatrics, Center for Vaccine Development and Global Health, University of Maryland School of Medicine, Baltimore, MD, USA
15. Centre pour le Développement des Vaccines, Bamako, Mali
16. Department of Pediatrics and Child Heath, Aga Khan University, Karachi, Pakistan

Confidential: For Review Only

17. Department of Maternal, Child, and Adolescent Health and Aging, World Health Organization, Geneva, Switzerland
18. Departments of Global Health and Population and Nutrition, Harvard T.H. Chan School of Public Health, Boston, Massachusetts
19. Division of Gastroenterology, Hepatology and Nutrition, Boston Children's Hospital, Department of Nutrition, Harvard T.H. Chan School of Public Health, Boston, Massachusetts

Keywords: bacterial diarrhea, molecular diagnostics, pediatric diarrhea, nutritional status

Confidential: For Review Only

ABSTRACT

Objective: The objective was to assess the association between nutritional and clinical characteristics and qPCR-diagnosis of bacterial diarrhea in a multicenter cohort of children under two years of age with moderate to severe diarrhea (MSD).

Design: A secondary cross-sectional analysis of baseline data collected from the AntiBiotics for Children with Diarrhea trial (NCT03130114).

Patients: Children with MSD (defined as ≥ 3 loose stools within 24 hours and presenting with at least one of the following: some/severe dehydration, moderate acute malnutrition (MAM), or severe stunting) enrolled in the ABCD trial and collected stool sample.

Study period: June 2017 to July 2019

Interventions: None

Main outcome measures: Likely bacterial etiology of diarrhea. Secondary outcomes included specific diarrhea etiology.

Results: A total of 6,692 children with MSD had qPCR results available and 28% had likely bacterial diarrhea etiology. Compared to children with severe stunting, children with MAM (adjusted odds ratio (aOR) [95% confidence intervals] 1.56 [1.18,2.08]), some/severe dehydration (aOR [95% CI] 1.66 [1.25,2.22]) or both (aOR [95% CI] 2.21 [1.61,3.06]), had higher odds of having likely bacterial diarrhea etiology. Similar trends were noted for ST-ETEC etiology. Clinical correlates including fever and prolonged duration of diarrhea were not associated with likely bacterial etiology; children with more than 6 stools in the previous 24h had higher odds of likely bacterial diarrhea (aOR [95% CI] 1.20 [1.05,1.36]) compared to those with fewer stools.

Confidential: For Review Only

Conclusion: The presence of MAM, dehydration or high stool frequency may be helpful in identifying children with MSD who might benefit from antibiotics.

What is already known on this topic

- Nutritional status has a bidirectional relationship with diarrhea incidence and severity.
- Clinical factors such as dehydration and prolonged duration are known to be a risk factor for severe diarrhea.
- Limited data exist on the relationship between nutritional and clinical status and specific infectious etiology of childhood diarrhea.

What this study adds

- Moderate acute malnutrition, dehydration, or both were found to have increased odds of likely bacterial etiology as compared to severe stunting among children with moderate to severe diarrhea.
- Children with more than 6 loose or watery stools in the 24h before presentation are at higher odds of likely bacterial diarrhea compared to those with lower stool frequency.

How this study might affect research, practice or policy

- Identifying children at high risk of bacterial diarrhea may allow a targeted approach with antibiotics during an acute diarrheal episode.

Confidential: For Review Only

INTRODUCTION

Diarrhea causes over 400,000 deaths annually and strategies to reduce this burden are informed by studies of diarrhea etiology (1).Bacterial pathogens including enterotoxigenic *Escherichia coli (E.coli)* encoding heat stable toxin (ST-ETEC) and *Shigella* are commonly associated with childhood diarrhea (2,3). The World Health Organization (WHO) recommends antibiotics for diarrhea only if there is blood in stool or in suspected cases of cholera(4). Increasing evidence, however, suggest that acute watery diarrhea can have bacterial (*Shigella* or enteroinvasive *Escherichia coli* EIEC) etiology as seen in an age-stratified case-control global enteric multicenter study (GEMs). This study enrolled cases who had moderate to severe diarrhea in children under 5 and noted in their collected stool samples that 40.3% *Shigella* spp or EIEC diarrhea attributed cases were non-dysenteric(3). Whether children with diarrhea of bacterial etiology can be identified, may have implications for antibiotic treatment recommendations.

While the relationship between nutritional status and incidence of diarrheal disease is well established, few studies have examined the relationship between child anthropometric and clinical characteristics with the specific etiology of diarrhea. Among 2 to 5-year-old Bangladeshi children with diarrhea, those with malnutrition (defined as weight-for-age Z-score < -2) were more likely to present with specific parasitic and bacterial enteropathogens specifically enterotoxigenic *Escherichia coli* (ETEC) (5). Another study in Kenya noted that malnourished children were more likely to have moderate to severe diarrhea due to enteroaggregative *Escherichia coli* (EAEC) but in another study in Bangladesh in a similar cohort, similar children were likely to present with *Shigella*(6,7). While these studies suggest that acute malnutrition may be an important risk factor for bacterial causes of diarrhea, they are notable for being performed at single sites, among children with either low-risk diarrhea or in hospitalized children, or collected with traditional bacterial culture methods, and/or for a limited number of pathogens. We recently completed a large, multicenter clinical trial of antibiotics for moderate-to-severe diarrhea in children ages 2-23 months (8,9). This cohort provided an opportunity to better understand whether

Confidential: For Review Only

nutritional status and other clinical characteristics were associated with specific pathogens causing diarrhea across several countries. Participants in the trial presented with moderate-to-severe diarrhea (MSD) and were assessed using molecular detection tools for several types of enteropathogens. We hypothesized that among children with MSD, those with MAM would be more likely to have a bacterial etiology.

METHODS

Participants

We studied a subsample of participants from the ABCD trial, a randomized, blinded, placebo-controlled, multicountry trial designed to evaluate whether azithromycin would reduce all-cause mortality in young children with undernutrition and MSD (8,9). The trial was implemented in Bangladesh, India, Kenya, Malawi, Mali, Pakistan and Tanzania between June 2017 and July 2019 in MSD children 2-23 months of age. For the purpose of this study, MSD was defined as a history of acute watery diarrhea (caretaker report of $\geq$ 3 loose/watery stool in the past 24 hours) for <14 days with one of more of the following: the presence of some/severe dehydration per WHO standards(10) MAM (defined as weight-for-length z-score [WLZ] $\leq$ -2 and > -3 (or mid-upper arm circumference [MUAC] $\geq$ 115 mm and < 125 mm for children over 6 months)); or severe stunting (length-for-age z-score [LAZ] < -3). Exclusion criteria included receipt of antibiotics currently/ in previous 14 days; allergy/contraindication to azithromycin; had dysentery; severe acute malnutrition or clinical suspicion of *V. cholerae* infection; previous/current enrollment of the child in this/any interventional trial; enrollment of a sibling or another child in the same household; or living at a distance from the enrolment center that would prevent direct observation on days 2 and 3. After written informed consent was obtained, participants were randomized to receive either a three day course of azithromycin or placebo (9). The subsample were participants identified randomly, at baseline with equal numbers from all sites.

Confidential: For Review Only

Exposure and Confounding Variables

MSD defining characteristics were the primary exposure of interest and included severe stunting, MAM, some/severe dehydration, or a combination of either of the two or all three characteristics. All anthropometric measurements were performed by 2 independant trained assessors according to WHO guidelines (8). Weight was measured using an electronic scale to the closest $\pm$ 10 g, length was obtained using a length board to the nearest 0.1 cm and MUAC was obtained using a non-stretchable tape to the nearest 0.1 cm. Study physicians measured the temperature using a digital thermometer at screening. Fever was calculated as temperature $\geq 37.5^{o}$C. Diarrhea duration and diarrhea frequency in the past 24 hours was recorded from mothers recall. Numerous confounding variables were collected at baseline using standardized questionnaires across all sites (Supplementary figure 1). A wealth quintile variable was created using country specific wealth distribution as reported in each country's most recent Demographic and Health Survey (11).

Outcome Variables

The primary outcome variable was likely bacterial diarrhea etiology assessed by qPCR on a baseline stool sample. Secondary outcomes included specific bacterial diarrhea etiology (likely ST-ETEC or *Shigella)*. Stool or rectal swab samples were tested by qPCR with a customized 85-target TaqMan array card to determine a cycle threshold (CTt) value and is detailed elsewhere (3). Pathogen-specific cutoffs were developed to assign likely diarrhea etiology based on the quantity of pathogen DNA/RNA in the stool sample (i.e., pathogen burden). These cut-offs(12) were obtained using adapted statistical models from two previously performed large multisite diarrhea studies: the seven-site Global Enteric Multicenter Study (GEMS) and the eight-site Malnutrition and the Consequences for Child Health and Development (MAL-ED) cohort study (3,13). Each cut-off was calculated by taking the median quantity-specific odds ratio from site specific models from previous studies. Then, the episode-specific attributable fraction (AFe) was

Confidential: For Review Only

calculated. Finally, a LOESS regression was fit and the highest Ct value with ≥ 0.5 Afe value was picked as the cut-off for each pathogen (14,15).

For this analysis, Ct values lower than these cut-offs were considered as "likely" bacterial diarrhea associated etiology. Specimens assessed as "likely" were compared with the other category. Ascribing etiology to "likely" with these cut-offs has been previously performed (16). Bacteria assessed for etiology and their Ct cut-off values included *Campylobacter* (16.3), tEPEC (18.1), ST-ETEC (25.4), *Salmonella* (31.9), *Shigella* (28.7), *and V.cholerae* (32.6). *Shigella* and ST-ETEC were chosen as the main specific etiologies since they comprised of about 84% of all bacterial enteropathogens. Due to logistical and budgetary constraints, stool/rectal swabs from approximately the first 1,000 participants at each site were collected. Whole stool sample or flocked rectal swab were stored at -80˚C until analysis.

Statistical Analysis

We conducted a cross-sectional analysis of baseline data from the ABCD trial. Logistic regressions were used to examine the association of clinical and anthropometric characteristics, including MSD defining characteristics: fever, diarrhea duration, and stool frequency, with likely bacterial diarrhea etiology. For the variable MSD defining characteristic, the presence of severe stunting alone was used as the reference category since participants with severe stunting had the lowest prevalence of likely bacterial diarrhea. Some previous studies also suggest that stunting is not associated with presence of specific pathogens in stools of children with diarrhea (7,17).

Diarrhea duration at enrollment was categorized as 0-6 days of diarrhea versus prolonged diarrhea of 7-14 days, and diarrhea frequency in the past 24 hours was categorized as low frequency (3-6 stools) versus high frequency (>6 stools). Bivariate(unadjusted) and multivariable analyses were conducted. Multivariable models included covariates for potential confounding factors that may be associated with both child nutritional status and diarrhea etiology based on a literature review. All

Confidential: For Review Only

statistical analyses were performed using R version 4.0.2 Software (R Foundation for Statistical Computing).

RESULTS

The parent ABCD trial enrolled 8,268 participants who presented with MSD. In total, 6,699 had stool/rectal samples collected at baseline and qPCR testing done,. Of those, 2 had missing/invalid qPCR results and 5 had no bacterial diarrhea etiology. Upon recalculation of anthropometric indexes, seven participants were deemed to have been erroneously enrolled, as they did not meet all enrolment criteria. The deviations from anthropometric enrolment cut-offs were, however, minor and these seven children were retained in the sample. Therefore, 6,692 participants across the study sites were included in this analysis (Figure 1) and 28% overall had diarrhea of likely bacterial etiology (Table 1).

We first examined the association of MSD-defining characteristics with likely bacterial etiology. Compared to children with severe stunting and adjusting for multiple clinical and demographic factors, children presenting with some/severe dehydration only (adjusted odds ratio 1.67 [95% confidence intervals [1.26, 2.24]), MAM (aOR 1.57 [1.19,2.09]), and both MAM and dehydration (aOR 2.23 [1.62,3.08]) were significantly at higher odds of a likely bacterial infection (Table 2). A similar trend was noted for ST-ETEC etiology where children presenting with dehydration (aOR 1.74 [1.19,2.62]), MAM (aOR 1.69 [1.15,2.53]), and MAM and dehydration (aOR 1.83 [1.19,2.89]) compared to those severely stunted, were at higher odds of a likely ST-ETEC etiology (Table 3). For *Shigella*, only children presenting with MAM and dehydration were found to have higher odds (aOR 1.64 [1.09,2.50]) compared to those severely stunted (Table 3). In bivariate associations of likely bacterial etiology, MAM and dehydration increased odds of likely bacterial etiology (OR 1.70 [1.28,2.27]) and MAM increased the odds of likely ST-ETEC etiology (OR 1.58 [1.13,2.26]) versus severe stunting (Supplementary table 1).

Confidential: For Review Only

Fever and duration of diarrhea were not associated with bacterial etiology or *Shigella* or ST-ETEC etiology (Table 2 & 3). However, children with high stool frequency (>6 stools) had increased odds of likely bacterial etiology (aOR 1.20 [1.05,1.36]) compared to those with lower stool frequency.

Since the qPCR analysis often detected more than one type of enteropathogen, we performed sensitivity analyses to evaluate the association between clinical and anthropometric characteristics with likely bacterial diarrhea etiology in children where only the enteropathogen of interest was found. The proportion of children who had only a single likely diarrhea bacterial etiology was 1,110/6,648(16.7%). No statistically significant associations were observed between anthropometrics and specific likely bacterial pathogens in these sensitivity analyses. High stool frequency, however, was associated with higher odds of likely bacterial diarrhea etiology (aOR 1.23 [1.05,1.45]), and *Shigella* etiology (aOR 1.32 [1.04,1.68]) after adjusting for multiple factors (Supplementary Tables 2 and 3).

DISCUSSION

In this analysis of 6,692 children with MSD whose etiology was assessed by qPCR methods, we found that several anthropometric and clinical factors were associated with likely bacterial diarrhea etiology. Children presenting with either MAM, dehydration or a combination of both, had higher odds of a likely bacterial and ST-ETEC diarrhea etiology compared to severely stunted children. Children with MAM and dehydration were also more likely to have *Shigella* diarrhea etiology compared to severely stunted children. Children with high stool frequency had higher odds of likely bacterial diarrhea etiology, a finding confirmed on sensitivity analysis evaluating children with only a single pathogen identified.

Our findings of association between MAM and a likely bacterial and ST-ETEC diarrhea etiology are consistent with some earlier studies (7,18). Tickell *et al*. studied 1,363 Kenyan children 6-59 months old who presented with acute diarrhea. The study noted that children with acute diarrhea and wasting (defined as MUAC <12.5 cm) were 1.8 times more likely to have enteroaggregative *E. coli* diarrhea etiology

Confidential: For Review Only

compared to those not wasted. Although the definition of acute malnutrition was different from our definition, they used similar PCR-based automated bacterial pathogen detection methods. A case-control study among 6-23 month old children in Bangladesh(18) noted that bacterial enteropathogens such as *Shigella* and EAEC were more common among children who were underweight (defined as WAZ<-2), a finding similar to ours. This study, however, enrolled children with malnutrition and only ~5% of those enrolled had diarrhea at baseline, whereas in our study all children had MSD. Similarly, a recent cross-sectional study carried out in Ethiopia noted that being wasted was associated with Salmonella infection (19).

Children with some/severe dehydration, with/without MAM, had greater odds of likely bacterial or ST-ETEC diarrhea etiology compared to children with severe stunting. Some/severe dehydration is known to be a risk factor for severe diarrhea (and even death) in children but not usually associated with bacterial diarrhea (20,21). In a study carried out in chidren <5 years presenting to a health clinic in Rwanda, *Shigella* was not associated with severe dehydration(22). Moreover, in a retrospective study carried out in Lao PDR to evaluate clinical and etiology associated with severity of acute diarrhea, among hospitalized children, 48.4% presented with dehydration and prevalence of dehydration was higher in those with rotavirus etiology versus rotavirus negative children. This association of dehydration was not noted in children with *Shigella* or *Salmonella* etiology, although the low numbers of samples positive for *Salmonella*(n=4) and *Shigella*(n=1) versus rotavirus (n=10) is noteworthy (23). Neither of the studies noted the association of dehydration in any bacterial diarrhea in contrast with our findings.

A re-analysis of the ABCD trial including molecular diagnostics was conducted to understand the effect of azithromycin in the outcomes in children with and without bacterial diarrhea etiology (16). It was noted that among those with likely and possible bacterial etiology, those in the azithromycin arm had lower risk of diarrhea at 3-days after randomization in comparison to those in the placebo arm.

Confidential: For Review Only

Additionally, within the 90-day follow-up period, azithromycin use was associated with lower risk of deaths/hospitalizations in children with likely and possible bacterial diarrhea. As noted in Pavlinac et al.'s results and previous studies, bacterial diarrhea may not always present with clinical signs and symptoms of dysentery (13,24) and therefore, limiting antibiotics to those with clinically apparent dysentery may limit treatment of bacterial diarrhea. The findings from our analysis outlines clinical and nutritional risk factors to potentially identify such high risk children who may benefit from antibiotics before culture results are known.

Our secondary analysis has some limitations. First, the observational, cross-sectional nature of the analysis limits the ability to make causal inferences. Second, we may not have had adequate power to detect more moderate magnitudes of association between nutritional status and diarrhea etiology. Third, our study included children with MSD which may limit the generalizability of our findings. We note that lack of a non-malnourished group (or severely malnourished group such as SAM) adds to the limited of generalizability. Finally, the use of enteropathogens cut-offs is complicated in settings where asymptomatic carriage of enteropathogens is high. It is noteworthy that these cut-offs were derived from previous case-control studies, thus may be subject to further refinement. It is also noted that there were a small number of children with individual pathogens which limited the power to detect differences between groups. Strengths of the study include the multi-country setting, the prospective nature of the data collection, the inclusion of numerous possible confounding variables, standardized measures of anthropometric data, and the use of novel enteropathogen detection tools. We also note that additional co-variates that could influence the associations such as pre-term birth, bloody diarrhea, severe wasting were not included since some were not measured and others were exclusion criteria..

In summary, in this large cohort of children with MSD, we note that moderate acute malnutrition and dehydration as well as high frequency of stools were associated with an increased odds of likely

Confidential: For Review Only

bacterial etiology, compared to those with severe stunting. These findings suggest that these characteristics could be helpful criteria for children presenting with moderate to severe diarrhea who might benefit from antibiotics during an acute diarrheal episode. Future studies could examine other, more specific risk factors for diarrhea etiology, including micronutrient status (25,26), exposure to food and water sources (27–29), vaccine status (30) and others. Additional trials of the utility of targeted antibiotic therapy also seem warranted.

Acknowledgements: We would like to thank the families and children who participated in this trial as well as the staff who helped carried out the trial in each of the 7 sites. We also thank additional members of the ABCD Study Group: Bangladesh: Muhammad Waliur Rahman, Irin Parvin, and Md. Farhad Kabir. India: Pratibha Dhingra, Arup Dutta, Anil Kumar Sharma, Vijay Kumar Jaiswal. Kenya: Churchil Nyabinda, Christine McGrath, Emily L. Deichsel, Maurine Anyango, Kevin Mwangi Kariuki, Doreen Rwigi, and Stephanie N. Tornberg-Belanger. Mali: Fadima Cheick Haidara, Flanon Coulibaly, Jasnehta Permala-Booth, and Dramane Malle. Malawi: Nigel Cunliffe, Latif Ndeketa, Desiree Witte, Chifundo Ndamala. Pakistan: Shahida Qureshi, Sadia Shakoor, Rozina Thobani, and Jan Mohammed. Tanzania: Rodrick Kisenge, Mohamed Bakari, Cecylia Msemwa, and Abraham Samma. TAC: James Platts-Mills and Jie Liu.

Contributors: SS, PA, KM, CD, CS: Conceptualization. All authors: Approval and Validation of concept. SS, PA, KP, CD, CS: Methodology. SS, CR – Analysis. SS, CD: Writing – original draft. All authors: Critical review, comments and edits of paper. PA, KM, CD: Supervision

Funding: The parent trial was funded by the Bill and Melinda Gates Foundation (grant OPP 1126331). Under the grant conditions of the Foundation, a Creative Commons Attribution 4.0 Generic License has already been assigned to the Author Accepted Manuscript version that might arise from this submission. The study sponsor had no role in the analysis, interpretation of results, writing of this article or decision

Confidential: For Review Only

to submit this paper for publication. CPD was supported, in part, by the National Institutes of Health (K24 DK104676 and P30 DK040561)

Competing interests: None declared

Patient Consent for publication: Not applicable

Ethics approval: The ABCD trial (ClinicalTrials.gov Identifier: NCT03130114) was approved by the WHO Ethics Review Committee and the institutional review boards of all participating sites; Bangladesh (International Centre for Diarrhoeal Disease Research), India (Institutional Ethics Committee, Subharti Medical College & Hospital, Swami Vivekan and Subharti University), Kenya (Kenya Medical Research Institute Ethical Review Committee and the University of Washington Institutional Review Board), Malawi (University of Malawi College of Medicine Research Ethics Committee), Mali (Comite d' Ethiquedela FMPOS (ERCatUSTTB)), Pakistan (Aga Khan University, Ethical Review Committee), Tanzania (Muhimbili University of Health and Allied Sciences Senate Research and Publications Committee; Tanzanian Food and Drug Administration; National Institute for Medical Research), and United States (Boston Children's Hospital Institutional Review Board; University of Maryland, Baltimore Research Ethics Committee).

Data availability statement: The de-identified patient data are available upon reasonable request.

Confidential: For Review Only

REFERENCES:

1. Troeger C, Blacker BF, Khalil IA, Rao PC, Cao S, Zimsen SR, et al. Estimates of the global, regional, and national morbidity, mortality, and aetiologies of diarrhoea in 195 countries: a systematic analysis for the Global Burden of Disease Study 2016. Lancet Infect Dis. 2018 Nov 1;18(11):1211–28.

2. Nuzhat S, Shahunja KM, Shahid ASMSB, Khan SH, Islam SB, Islam MR, et al. Diarrhoeal children with concurrent severe wasting and stunting compared to severe wasting or severe stunting. Trop Med Int Health. 2020;25(8):928–35.

3. Liu J, Platts-Mills JA, Juma J, Kabir F, Nkeze J, Okoi C, et al. Use of quantitative molecular diagnostic methods to identify causes of diarrhoea in children: a reanalysis of the GEMS case-control study. Lancet Lond Engl. 2016 Sep 24;388(10051):1291–301.

4. World Health Organization, Department of Child and Adolescent Health and Development. The treatment of diarrhoea: a manual for physicians and other senior health workers. Geneva: Dept. of Child and Adolescent Health and Development, World Health Organization; 2005.

5. Mondal D, Haque R, Sack RB, Kirkpatrick BD, Petri WA. Attribution of Malnutrition to Cause-Specific Diarrheal Illness: Evidence from a Prospective Study of Preschool Children in Mirpur, Dhaka, Bangladesh. Am J Trop Med Hyg. 2009 May;80(5):824–6.

6. Ferdous F, Das SK, Ahmed S, Farzana FD, Latham JR, Chisti MJ, et al. Severity of Diarrhea and Malnutrition among Under Five-Year-Old Children in Rural Bangladesh. Am J Trop Med Hyg. 2013 Aug 7;89(2):223–8.

7. Tickell KD, Pavlinac PB, John-Stewart GC, Denno DM, Richardson BA, Naulikha JM, et al. Impact of Childhood Nutritional Status on Pathogen Prevalence and Severity of Acute Diarrhea. Am J Trop Med Hyg. 2017 Nov;97(5):1337–44.

8. ABCD study team. A double-blind placebo-controlled trial of azithromycin to reduce mortality and improve growth in high-risk young children with non-bloody diarrhoea in low resource settings: the Antibiotics for Children with Diarrhoea (ABCD) trial protocol. Trials. 2020 Jan 13;21(1):71.

9. The Antibiotics for Children With Diarrhea (ABCD) Study Group. Effect of 3 Days of Oral Azithromycin on Young Children With Acute Diarrhea in Low-Resource Settings: A Randomized Clinical Trial. JAMA Netw Open. 2021 Dec 16;4(12):e2136726.

10. Pocket book of hospital care for children: Second edition [Internet]. [cited 2024 Feb 19]. Available from: https://www.who.int/publications/i/item/978-92-4-154837-3

11. Colston JM, Ahmed AMS, Soofi SB, Svensen E, Haque R, Shrestha J, et al. Seasonality and within-subject clustering of rotavirus infections in an eight-site birth cohort study. Epidemiol Infect. 2018 Apr;146(6):688–97.

Confidential: For Review Only

12. Pavlinac PB, Platts-Mills J, Liu J, Atlas HE, Gratz J, Operario DJ, et al. Effect of azithromycin on bacterial acute watery diarrhea: A re-analysis of the AntiBiotics for Children with severe Diarrhea (ABCD) trial incorporating molecular diagnostics. J Infect Dis.

13. Platts-Mills JA, Liu J, Rogawski ET, Kabir F, Lertsethtakarn P, Siguas M, et al. Use of quantitative molecular diagnostic methods to assess the aetiology, burden, and clinical characteristics of diarrhoea in children in low-resource settings: a reanalysis of the MAL-ED cohort study. Lancet Glob Health. 2018 Dec;6(12):e1309–18.

14. Kotloff KL, Blackwelder WC, Nasrin D, Nataro JP, Farag TH, van Eijk A, et al. The Global Enteric Multicenter Study (GEMS) of Diarrheal Disease in Infants and Young Children in Developing Countries: Epidemiologic and Clinical Methods of the Case/Control Study. Clin Infect Dis Off Publ Infect Dis Soc Am. 2012 Dec 15;55(Suppl 4):S232–45.

15. The MAL-ED Network Investigators. The MAL-ED Study: A Multinational and Multidisciplinary Approach to Understand the Relationship Between Enteric Pathogens, Malnutrition, Gut Physiology, Physical Growth, Cognitive Development, and Immune Responses in Infants and Children Up to 2 Years of Age in Resource-Poor Environments. Clin Infect Dis. 2014 Nov 1;59(suppl_4):S193–206.

16. Pavlinac PB, Platts-Mills JA, Liu J, Atlas HE, Gratz J, Operario D, et al. Azithromycin for Bacterial Watery Diarrhea: A Reanalysis of the AntiBiotics for Children With Severe Diarrhea (ABCD) Trial Incorporating Molecular Diagnostics. J Infect Dis. 2023 Jul 5;jiad252.

17. Kotloff KL, Nataro JP, Blackwelder WC, Nasrin D, Farag TH, Panchalingam S, et al. Burden and aetiology of diarrhoeal disease in infants and young children in developing countries (the Global Enteric Multicenter Study, GEMS): a prospective, case-control study. The Lancet. 2013 Jul 20;382(9888):209–22.

18. Platts-Mills JA, Taniuchi M, Uddin MJ, Sobuz SU, Mahfuz M, Gaffar SA, et al. Association between enteropathogens and malnutrition in children aged 6–23 mo in Bangladesh: a case-control study. Am J Clin Nutr. 2017 May 1;105(5):1132–8.

19. Balew M, Kibret M. Prevalence of enteric bacterial pathogens in diarrheic under-five children and their association with the nutritional status in Bahir Dar Zuria District, Northwest Ethiopia. BMC Nutr. 2023 Feb 24;9(1):35.

20. Griffin PM, Ryan CA, Nyaphisi M, Hargrett-Bean N, Waldman RJ, Blake PA. Risk factors for fatal diarrhea: a case-control study of African children. Am J Epidemiol. 1988 Dec;128(6):1322–9.

21. O'Reilly CE, Jaron P, Ochieng B, Nyaguara A, Tate JE, Parsons MB, et al. Risk factors for death among children less than 5 years old hospitalized with diarrhea in rural western Kenya, 2005-2007: a cohort study. PLoS Med. 2012;9(7):e1001256.

22. Kabayiza JC, Andersson ME, Nilsson S, Baribwira C, Muhirwa G, Bergström T, et al. Diarrhoeagenic microbes by real-time PCR in Rwandan children under 5 years of age with acute gastroenteritis. Clin Microbiol Infect. 2014 Dec 1;20(12):O1128–35.

Confidential: For Review Only

23. Vorlasane L, Luu MN, Tiwari R, Imoto A, Sato M, Huy NT, et al. The clinical characteristics, etiologic pathogens and the risk factors associated with dehydration status among under-five children hospitalized with acute diarrhea in Savannakhet Province, Lao PDR. PLOS ONE. 2023 Mar 2;18(3):e0281650.

24. Tickell KD, Brander RL, Atlas HE, Pernica JM, Walson JL, Pavlinac PB. Identification and management of Shigella infection in children with diarrhoea: a systematic review and meta-analysis. Lancet Glob Health. 2017 Dec 1;5(12):e1235–48.

25. Khalili B, Hart A, Mardani M, Khalili M, McArdle F, Cuevas L. Diarrhea-associated micronutrient deficiencies and risk of subsequent diarrhea in admitted children to Hajar hospital in Shahrekord, Iran. Arch Clin Infect Dis [Internet]. 2007 [cited 2023 Mar 7]; Available from: https://www.semanticscholar.org/paper/Diarrhea-associated-micronutrient-deficiencies-and-Khalili-Hart/452eb0a4188665e4003d25c2dab055720b2262e4

26. Talachian E, Bidari A, Noorbakhsh S, Tabatabaei A, Salari F. Serum levels of vitamins A and D, and zinc in children with acute diarrhea: A cross-sectional study. Med J Islam Repub Iran. 2015 Apr 27;29:207.

27. George CM, Perin J, Neiswender de Calani KJ, Norman WR, Perry H, Davis TP, et al. Risk Factors for Diarrhea in Children under Five Years of Age Residing in Peri-urban Communities in Cochabamba, Bolivia. Am J Trop Med Hyg. 2014 Dec 3;91(6):1190–6.

28. Maponga BA, Chirundu D, Gombe NT, Tshimanga M, Shambira G, Takundwa L. Risk factors for contracting watery diarrhoea in Kadoma City, Zimbabwe, 2011: a case control study. BMC Infect Dis. 2013 Dec 2;13(1):567.

29. Knight SM, Toodayan W, Caique WC, Kyi W, Barnes A, Desmarchelier P. Risk Factors for the Transmission of Diarrhoea in Children: A Case-Control Study in Rural Malaysia. Int J Epidemiol. 1992 Aug 1;21(4):812–8.

30. Bahartha AS, AlEzzi JI. Risk factors of diarrhea in children under 5 years in Al-Mukalla, Yemen. Saudi Med J. 2015 Jun;36(6):720–4.

Confidential: For Review Only

Table 1: Baseline characteristics of 6,692 mothers and their 2-23-month-old children who presented with moderate-to-severe diarrhea.

Baseline characteristics	Bangladesh (n=1000)	India (n=998)	Pakistan (n=997)	Kenya (n=1014)	Malawi (n=691)	Mali (n=1000)	Tanzania (n=997)	ALL SITES (n=6,692)
Age (months)	11.2 (5.0)[*]	11.9 (5.6)[*]	12.5 (5.5)[*]	11.0 (5.7)[*]	12.0 (4.8)[*]	12.0 (4.6)[*]	11.0 (5.2)[*]	11.6 (5.3)[*]
Males	57.8%	52.9%	50.6%	52.7%	56.9%	53.9%	53.2%	53.8%
Any rotavirus vaccination	1.0%	2.7%	7.3%	99.6%	99.6%	97.6%	99.9%	60%
Exclusively breastfed at time of enrolment	1.7%	6.9%	9.2%	17.9%	4.1%	5.4%	14.4%	8.7%
Maternal age, years	24 (5)	26 (4)	27 (6)	26 (6)	25 (6)	26 (6)	27 (6)	26 (6)
Maternal body mass index, kg/m2	22.3 (4.3)	22.6 (4.3)	23.2 (5.0)	23.2 (3.8)	23.1 (5.3)	24.0 (4.9)	24.5 (5.0)	23.3 (4.7)
Maternal education, completed school years	5.8 (4.0)	4.8 (4.8)	2.9 (4.0)	9.0 (2.9)	8.3 (2.8)	4.3 (5.0)	7.8 (2.7)	6.0 (4.4)
Number of children <5 years of age in house	1.2 (0.4)	1.9 (0.9)	2.0 (1.0)	1.7 (0.8)	1.4 (0.6)	2.4 (1.8)	1.3 (0.5)	1.7 (1.1#)
Fever (defined as temperature $>37.5^{o}$C)	15.3%	7.6%	9.0%	6.0%	19.4%	16.9%	12.5%	12.1%
Some/severe dehydration	25.1%	48.4%	28.5%	91.8%	77.7%	23.1%	92.8%	54.4%
Length for age z-score (LAZ)	-1.83 (1.15)	-2.12 (1.28)	-2.15 (1.28)	-0.81 (1.26)	-1.55 (1.47)	-1.16 (1.13)	-0.76 (1.14)	-1.48 (1.36)
Weight for length z-score (WLZ)	-1.69 (0.90)	-1.37 (1.01)	-1.45 (0.96)	-0.28 (1.19)	-0.62 (1.19)	-2.04 (0.74)	-0.39 (1.27)	-1.14 (1.23)
Severe stunting[†]	19.5%	27.6%	27.5%	5.6%	16.5%	6.5%	2.2%	15.0%
Moderate Acute Malnutrition[‡]	73.6%	57.3%	70.6%	14.0%	26.0%	85.0%	16.1%	49.9%
Presence[§] of any bacterial pathogen[**] in stool	36.3%	27.5%	30.9%	21.5%	31.7%	28.6%	22.9%	28.3%
Presence[5] of Shigella in stool	12.8%	16.0%	18.2%	7.0%	11.3%	13.5%	9.2%	12.6%
Presence[5] of ST-ETEC[††] in stool	19.4%	9.9%	12.7%	10.4%	20.4%	11.9%	10.7%	13.3%

[*] Numbers indicate percentages for categorical variable and mean (SD) for continuous variables

[†] Defined as Length for age z-score of < -3.0

[‡] Defined as Weight for Length z-score < -2.0 & ≥-3.00 or Mid-upper arm Circumference of <12.5 & ≥ 11.5 if participant was 6 months or older

[§] Indicated as a Cq value below relevant cut-off in a quantitative polymerase chain reaction (Liu et at., 2016).

[**] Includes the following bacteria - Cq cut-off for diagnostic quantity to be termed as associated with diarrhea: Campylobacter spp (16.3), typical EPEC (18.1), ST-ETEC (25.4), Salmonella spp (31.9), Shigella spp (28.7), Vibrio cholerae (32.6)

[††] (Heat stable Toxin) STh-producing enterotoxigenic E. Coli

Confidential: For Review Only

Table 2: Clinical and nutritional correlates of likely bacterial etiology of diarrhea among 2-23-month-old children with moderate-to-severe diarrhea adjusted for factors

Bacterial etiology

	**Proportion n/N (%)**	**Adjusted* odds ratio (95% CI)**	**p-value**
**Moderate to severe diarrhea defining characteristics**			
**Severe stunting only**	94/417 (22.5%)	Ref.	-
**Some /severe dehydration only**	764/2,832 (27.0%)	1.67 (1.26, 2.24)	<0.001
**MAM only**	675/2,22[illegible]	[illegible]	0.002
**MAM and some /severe dehydration**	209/626 (33.4%)	2.23 (1.62, 3.08)	<0.001
**MAM and severe stunting**	109/408 (26.7%)	1.13 (0.79, 1.62)	0.52
**Some/severe dehydration and severe stunting**	22/95 (23.2%)	1.42 (0.79, 2.49)	0.23
**MAM, some/severe dehydration, and severe stunting**	20/84 (23.8%)	0.80 (0.39, 1.53)	0.52
**Fever**			
**No**	1,646/5,884 (28.0%)	Ref.	-
**Yes**	248/808 (30.7%)	1.11 (0.93, 1.32)	0.26
**Duration of diarrhea (excluding day of enrollment)**			
**Diarrhea (0-6 days)**	1,781/6,328 (28.1%)	Ref.	-
**Prolonged duration (7-14 days)**	113/364 (31.0%)	1.22 (0.94, 1.58)	0.13
**Frequency of diarrhea in the past 24 hours**			
**Low frequency (3-6 stools)**	912/3,536 (25.8%)	Ref.	-
**High frequency (>6 loose stools)**	982/3,156 (31.1%)	1.20 (1.05, 1.36)	0.007

	***Escherichia coli* encoding heat-stable toxin etiology**			***Shigella* etiology**		
	**Proportion n/N (%)**	**Adjusted[1] odds ratio (95% CI)**	**p-value**	**Proportion n/N (%)**	**Adjusted[1] odds ratio (95% CI)**	**p-value**
**Moderate to severe diarrhea defining characteristics**						
**Severe stunting only**	40/417 (9.6%)	Ref.	-	52/417 (12.5%)	Ref.	-
**Some/severe dehydration only**	378/2815 (13.4%)	1.74 (1.19, 2.62)	0.006	[illegible]	1.18 (0.82, 1.73)	0.38
**MAM only**	319/ 2,227 (14.3%)	1.69 (1.15, 2.53)	0.009	320/2224 (14.4%)	1.29 (0.90, 1.86)	0.17
**MAM and some /severe**	81/623 (13.0%)	1.83 (1.18, 2.89)	0.008	90/626 (14.4%)	1.64 (1.09, 2.50)	0.02

Adjusted for: child age, child sex, household wealth quintile, maternal age, maternal education, country of enrolment, rotavirus vaccination status, maternal BMI, total number of children under age 5 year in the household, defining characteristic of moderate to severe diarrhea, fever, duration of diarrhea, and high vs. low frequency of stools in past 24h.

Confidential: For Review Only

**dehydration**						
**MAM and severe stunting**	45/408 (11.0%)	1.04 (0.62,1.74)	0.87	63/408 (15.4%)	1.08 (0.69, 1.70)	0.75
**Some/severe dehydration and severe stunting**	14/95 (14.7%)	1.92 (0.92, 3.82)	0.07	5/95 (5.3%)	0.52 (0.17, 1.27)	0.19
**MAM, some/severe dehydration, and severe stunting**	12/84 (14.3%)	1.19 (0.46, 2.71)	0.69	10/84 (11.9%)	0.68 (0.25, 1.58)	0.41
**Fever**						
**No**	767/5,865 (13.1%)	Ref	-	736/5,883 (12.5%)	Ref	-
**Yes**	122/806 (15.1%)	1.12 (0.89, 1.40)	0.31	109/808 (13.5%)	1.15 (0.90, 1.46)	0.26
**Duration of diarrhea (excluding day of enrollment)**						
**Diarrhea (0-6 days)**	846/6,308 (13.4%)	Ref	-	783/6,327 (12.4%)	Ref	-
**Prolonged duration (7-14 days)**	43/363 (11.8%)	1.05 (0.72, 1.50)	0.79	62/364 (17.0%)	1.29 (0.92, 1.78)	0.13
**Frequency of diarrhea in the past 24 hours**						
**Low frequency (3-6 stools)**	433/3,521 (12.3%)	Ref	-	406/3,535 (11.5%)	Ref	-
**High frequency (>6 loose stools)**	456/3,150 (14.5%)	1.10 (0.93, 1.31)	0.28	439/3,156 (13.9%)	1.17 (0.98, 1.40)	0.08

Table 3: Clinical and nutritional correlates of likely *Escherichia coli* encoding heat-stable ST-ETEC and *Shigella* etiology of diarrhea among 2-23-month-old children with moderate-to-severe diarrhea adjusted for factors

Adjusted for: child age, child sex, household wealth quintile, maternal age, maternal education, country of enrolment, rotavirus vaccination status, maternal BMI, total number of children under age 5 year in the household, defining characteristic of moderate to severe diarrhea, fever, duration of diarrhea, and high vs. low frequency of stools in past 24h.

Figure 1: Flow chart of 2–23-month-old children presenting with moderate to severe diarrhea.

“Likely” diarrhea associated etiology was determined by Ct value cut-offs for specific enteropathogens. Ct values greater than these cut-offs but < 35 were considered “possible” etiologies while Ct value >35 was considered “Unlikely” etiology

Confidential: For Review Only

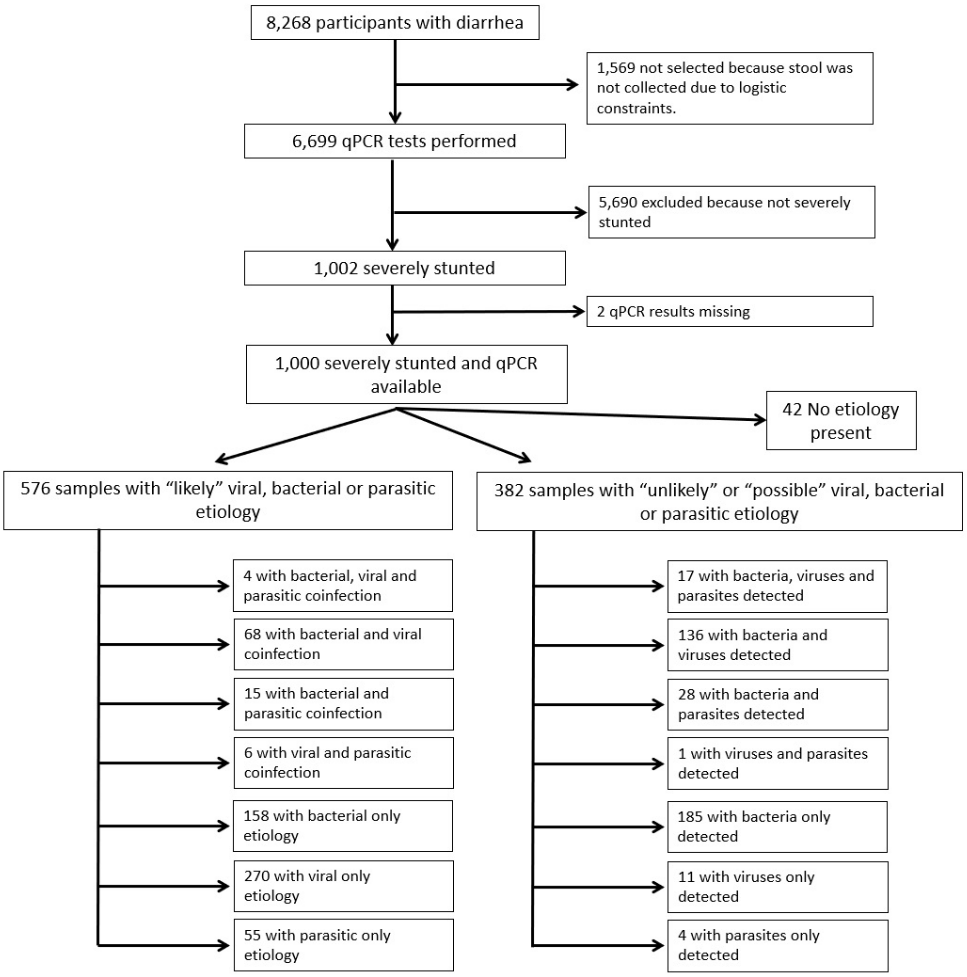

Figure 1: Flow chart of 2–23-month-old children presenting with moderate to severe diarrhea. "Likely" diarrhea associated etiology was determined by Ct value cut-offs for specific enteropathogens. Ct values greater than these cut-offs but < 35 were considered "possible" etiologies while Ct value >35 was considered "Unlikely" etiology

281x279mm (96 x 96 DPI)

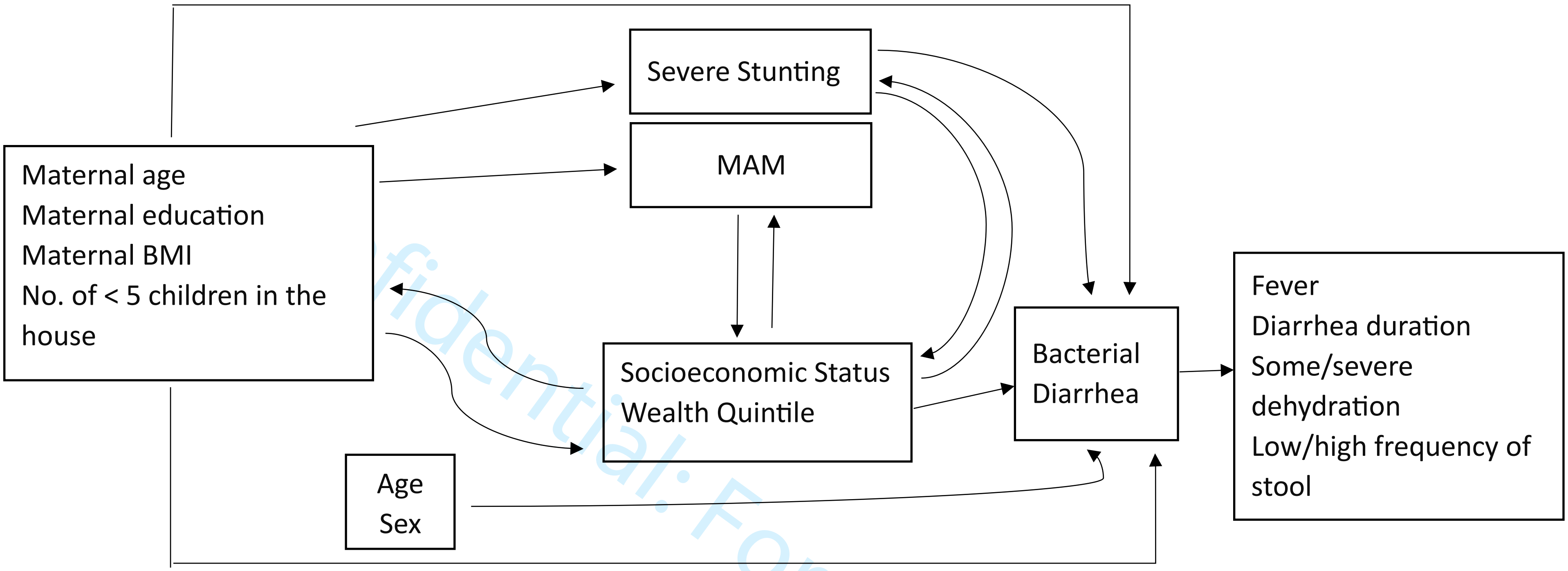

Supplementary Figure 1: A directed acyclic graph (DAG) depicting the relationship between the exposure, outcome and confounding variables that are included in this analysis.

Confidential: For Review Only

Supplementary table 1: Clinical and nutritional unadjusted odds ratios of likely bacterial etiology of diarrhea among 2-23-month-old children with moderate-to-severe diarrhea

	Bacterial etiology			Escherichia coli encoding heat-stable toxin etiology			Shigella etiology		
**Variable**	**Proportion n/N (%)**	**Unadjusted odds ratio (95% CI)**	**p-value**	**Proportion n/N (%)**	**Unadjusted odds ratio (95% CI)**	**p-value**	**Proportion n/N (%)**	**Unadjusted odds ratio (95% CI)**	**p-value**
**Moderate to severe diarrhea defining characteristics**									
**Severe stunting only**	94/417 (22.5%)	Ref	-	40/417 (9.6%)	Ref	-	52/417 (12.5%)	Ref	-
**Some / severe dehydration only**	764/2,832 (27.0%)	1.27 (1.00,1.63)	0.06	378/2815 (13.4%)	1.46 (1.05,2.09)	0.030	305/2832 (10.8%)	0.85 (0.62, 1.17)	0.30
**MAM only**	675/2,228 (30.3%)	1.47 (1.16,1.90)	0.93	319/ 2,227 (14.3%)	1.58 (1.13,2.26)	0.01	320/2224 (14.4%)	1.18 (0.87,1.63)	0.31
**MAM and some /severe dehydration**	209/626 (33.4%)	1.70 (1.28,2.27)	0.002	81/623 (13.0%)	1.41 (0.95,2.12)	0.09	90/626 (14.4%)	1.18 (0.82,1.71)	0.38
**MAM and severe stunting**	109/408 (26.7%)	1.24 (0.90,1.70)	0.19	45/408 (11.0%)	1.17 (0.75,1.84)	0.50	63/408 (15.4%)	1.28 (0.86,1.91)	0.22
**Some/severe dehydration and severe stunting**	22/95 (23.2%)	1.02 (0.59, 1.71)	0.93	14/95 (14.7%)	1.63 (0.82,3.07)	0.15	5/95 (5.3%)	0.39 (0.13,0.92)	0.051
**MAM, some/severe dehydration and severe stunting**	20/84 (23.8%)	1.06 (0.60,1.81)	0.84	12/84 (14.3%)	1.57 (0.76,3.06)	0.20	10/84 (11.9%)	0.95 (0.44, 1.88)	0.89
**Fever**									
**No**	1,646/5,884 (28.0%)	Ref	-	767/5,865 (13.1%)	Ref	-	736/5,883 (12.5%)	Ref	-
**Yes**	248/808 (30.7%)	1.14 (0.97,1.34)	0.11	122/806 (15.1%)	1.19 (0.96. 1.45)	0.11	109/808 (13.5%)	1.09 (0.88, 1.35)	0.43
**Duration of diarrhea (excluding day of enrollment)**									
**Diarrhea (0-6 days)**	1,781/6,328 (28.1%)	Ref	-	846/6,308 (13.4%)	Ref	-	783/6,327 (12.4%)	Ref	-
**Prolonged duration (7-14 days)**	113/364 (31.0%)	1.15 (0.91,1.44)	0.23	43/363 (11.8%)	0.87 (0.62, 1.19)	0.39	62/364 (17.0%)	1.45 (1.09,1.92)	0.010
**Frequency of diarrhea in the past 24 hours**									
**Low frequency (3-6 stools)**	912/3,536 (25.8%)	Ref	-	433/3,521 (12.3%)	Ref	-	406/3,535 (11.5%)	Ref	-
**High frequency (>6 loose stools)**	982/3,156 (31.1%)	1.30 (1.17, 1.45)	<0.001	456/3,150 (14.5%)	1.21 (1.05, 1.39)	0.009	439/3,156 (13.9%)	1.25 (1.08,1.44)	0.003

Confidential: For Review Only

Supplementary table 2: Clinical and nutritional correlates with likely bacterial diarrheal etiology with no coinfection including enterotoxigenic Escherichia coli (E.coli) encoding heat-stable toxin (ST-ETEC) and Shigella diarrheal etiology with no coinfection in 2-23-month-old children with moderate-to-severe diarrhea adjusted for factors.

	**Bacterial etiology (no coinfection)**			***Escherichia coli* encoding heat-stable toxin etiology (no coinfection)**			***Shigella* etiology (no coinfection)**		
	**Proportion n/N (%)**	**Adjusted[1] odds ratio (95% CI)**	**p-value**	**Proportion n/N (%)**	**Adjusted[1] odds ratio (95% CI)**	**p-value**	**Proportion n/N (%)**	**Adjusted[1] odds ratio (95% CI)**	**p-value**
**Moderate to severe diarrhea defining characteristics**									
**Severe stunting only**	65/417 (15.6%)	Ref.	-	18/417 (4.3%)	Ref.	-	35/417 (8.4%)	Ref.	-
**Some /severe dehydration only**	464/2,336 (16.6%)	1.34 (0.96, 1.88)	0.088	156/2,824 (5.5%)	1.36 (0.79, 2.52)	0.30	150/2,829 (5.3%)	0.66 (0.42, 1.05)	0.074
**MAM only**	382/2,2224 (17.2%)	1.18 (0.86,1.65)	0.31	116/2,221 (5.2%)	1.28 (0.74, 2.37)	0.40	154/2,223 (6.9%)	0.95 (0.61, 1.50)	0.81
**MAM and some /severe dehydration**	107/623 (17.2%)	1.38 (0.94, 2.02)	0.10	28/625 (4.5%)	1.23 (0.63, 2.47)	0.55	30/626 (4.8%)	0.64 (0.36, 1.14)	0.13
**MAM and severe stunting**	71/407 (17.4%)	0.91 (0.59, 1.39)	0.66	20/407 (4.9%)	1.05 (0.50, 2.22)	0.89	33/407 (8.1%)	0.61 (0.33, 1.11)	0.11
**Some/severe dehydration and severe stunting**	8/95 (8.4%)	0.70 (0.29, 1.47)	0.37	5/95 (5.3%)	1.38 (0.43, 3.75)	0.55	2/95 (2.1%)	0.28 (0.04, 0.98)	0.09
**MAM, some/severe dehydration, and severe stunting**	14/84 (16.7%)	0.75 (0.31, 1.60)	0.49	6/84 (7.1%)	1.40 (0.39, 4.05)	0.57	3/84 (3.6%)	0.29 (0.05, 1.01)	0.098
**Fever**									
**No**	986/5,845 (16.9%)	Ref.	-	312/5,870 (5.3%)	Ref	-	364/5,880 (6.2%)	Ref	-
**Yes**	124/803 (15.4%)	0.88 (0.70,1.10)	0.29	36/806 (4.5%)	0.79 (0.53, 1.15)	0.24	43/763 (5.3%)	0.88 (0.60,1.27)	0.52
**Duration of diarrhea (excluding day of enrollment)**									
**Diarrhea (0-6 days)**	1,036/ 6,285 (16.5%)	Ref.	-	329/6,314 (5.2%)	Ref	-	32/363 (8.8%)	Ref	-
**Prolonged duration (7-14 days)**	74/363 (20.4%)	1.22 (0.90,1.64)	0.19	19/362 (5.2%)	1.05 (0.60,1.73)	0.85	375/6,323 (5.9%)	1.19 (0.75,1.83)	0.44
**Frequency of diarrhea in the past 24 hours**									
**Low frequency (3-6 stools)**	534/3,508 (15.2%	Ref.	-	195/3,528 (5.5%)	Ref	-	202/3,534 (5.7%)	Ref	-
**High frequency (>6 loose stools)**	576/3,140 (18.3%)	1.23 (1.05,1.45)	0.0091	153/3,150 (4.9%)	0.99 (0.76, 1.28)	0.94	205/3,152 (6.5%)	1.32 (1.04,1.68)	0.025

[1] Adjusted for: child age, child sex, household wealth quintile, maternal age, maternal education, country of enrolment, rotavirus vaccination status, maternal BMI, total number of children under age 5 year in the household, defining characteristic of moderate to severe diarrhea, fever, duration of diarrhea, and high vs. low frequency of stools in past 24h.

Confidential: For Review Only

Supplementary table 3: Clinical and nutritional correlates with bacterial diarrheal etiology with no coinfection including enterotoxigenic Escherichia coli (E.coli) encoding heat-stable toxin (ST-ETEC) and Shigella diarrheal etiology with no coinfection in 2-23-month-old children with moderate-to-severe diarrhea.

	**Bacterial etiology (no coinfection)**			**Escherichia coli encoding heat-stable toxin etiology (no coinfection)**			**Shigella etiology (no coinfection)**		
**Variable**	**Proportion n/N (%)**	**Unadjusted odds ratio (95% CI)**	**p-value**	**Proportion n/N (%)**	**Unadjusted odds ratio (95% CI)**	**p-value**	**Proportion n/N (%)**	**Unadjusted odds ratio (95% CI)**	**p-value**
**Moderate to severe diarrhea defining characteristics**									
**Severe stunting only**	65/417 (15.6%)	Ref	-	18/417 (4.3%)	Ref.	-	35/417 (8.4%)	Ref	-
**Some / severe dehydration only**	464/2,336 (16.6%)	1.08 (0.82,1.44)	0.61	156/2,824 (5.5%)	1.30 (0.81,2.21)	0.31	150/2,829 (5.3%)	0.61 (0.42,0.91)	0.01
**MAM only**	382/2,2224 (17.2%)	1.12 (0.85, 1.51)	0.43	116/2,221 (5.2%)	1.22 (0.75,2.09)	0.45	154/2,223 (6.9%)	0.81 (0.56, 1.21)	0.28
**MAM and some /severe dehydration**	107/623 (17.2%)	1.12 (0.80,1.58)	0.50	28/625 (4.5%)	1.04 (0.57,1.94)	0.90	30/626 (4.8%)	0.55 (0.33,0.91)	0.020
**MAM and severe stunting**	71/407 (17.4%)	1.14 (0.79, 1.65)	0.48	20/407 (4.9%)	1.14 (0.59, 2.21)	0.69	33/407 (8.1%)	0.96 (0.58,1.58)	0.87
**Some/severe dehydration and severe stunting**	8/95 (8.4%)	0.50 (0.21, 1.02)	0.076	5/95 (5.3%)	1.23 (0.39, 3.18)	0.69	2/95 (2.1%)	0.24 (0.04, 0.79)	0.049
**MAM, some/severe dehydration and severe stunting**	14/84 (16.7%)	1.08 (0.56, 1.99)	0.80	6/84 (7.1%)	1.71 (0.60, 4.21)	0.27	3/84 (3.6%)	0.40 (0.09, 1.16)	0.14
**Fever**									
**No**	986/5,845 (16.9%)	Ref	-	312/5,870 (5.3%)	Ref	-	364/5,880 (6.2%)	Ref	-
**Yes**	124/803 (15.4%)	0.90 (0.73, 1.10)	0.31	36/806 (4.5%)	0.83 (0.58, 1.17)	0.31	43/763 (5.3%)	0.85 (0.61,1.17)	0.34
**Duration of diarrhea (excluding day of enrollment)**									
**Diarrhea (0-6 days)**	1,036/ 6,285 (16.5%)	Ref	-	329/6,314 (5.2%)	Ref	-	32/363 (8.8%)	Ref	-
**Prolonged duration (7-14 days)**	74/363 (20.4%)	1.30 (0.99,1.68)	0.05	19/362 (5.2%)	1.01 (0.61, 1.58)	0.98	375/6,323 (5.9%)	1.53 (1.03,2.20)	0.03
**Frequency of diarrhea in the past 24 hours**									
**Low frequency (3-6 stools)**	534/3,508 (15.2%	Ref	-	195/3,528 (5.5%)	Ref	-	202/3,534 (5.7%)	Ref	-
**High frequency (>6 loose stools)**	576/3,140 (18.3%)	1.25 (1.10,1.42)	<0.001	153/3,150 (4.9%)	0.87 (0.70, 1.08)	0.22	205/3,152 (6.5%)	1.15 (0.94, 1.40)	0.18

Confidential: For Review Only
